# Supplementary material for: Hypothetical Outcome Plots Outperform Error Bars and Violin Plots for Inferences about Reliability of Variable Ordering
Source: PLoS One. 2015 Nov 16;10(11):e0142444. doi: 10.1371/journal.pone.0142444 (PMC4646698; doi:10.1371/journal.pone.0142444)
Supplement: S1 File — This manuscript can be accessed as an animated PDF. View the PDF in Adobe Illustrator and click the plots to see the animated version. (PDF) [file pone.0142444.s001.pdf]

Hypothetical Outcome Plots
Outperform Error Bars and Violin Plots for Inferences About Reliability of Variable Ordering

Jessica Hullman[1,*], Paul Resnick[2], Eytan Adar[2],

1 Information School, University of Washington, Seattle, WA, USA
2 School of Information, University of Michigan, Ann Arbor, MI, USA

*** jhullman@uw.edu**

Abstract

Many visual depictions of probability distributions, such as error bars, are difficult for users to accurately interpret. We present and study an alternative representation, Hypothetical Outcome Plots (HOPs), that animates a finite set of individual draws. In contrast to the statistical background required to interpret many static representations of distributions, HOPs require relatively little background knowledge to interpret. Instead, HOPs enables viewers to infer properties of the distribution using mental processes like counting and integration. We conducted an experiment comparing HOPs to error bars and violin plots. With HOPs, people made much more accurate judgments about plots of two and three quantities. Accuracy was similar with all three representations for most questions about distributions of a single quantity.

Author Summary

It is important for users of visualizations, tables, and other data presentations to assess the uncertainty of inferred properties. Showing a probability distribution over possible outcomes is a common way to represent uncertainty. Many visual depictions of probability distributions, such as error bars, are difficult for users to accurately interpret. We present and study an alternative representation, Hypothetical Outcome Plots (HOPs), that animates a finite set of samples representing hypothetical outcomes. People can interpret and make estimates about uncertainty from HOPs without special training. In contrast to most static representations, HOPs enables viewers to infer properties of the distribution using mental processes like counting. We conducted an experiment comparing HOPs to error bars and violin plots. With HOPs, people made much more accurate judgments about plots of two and three quantities. Accuracy was similar with all three representations for most questions about distributions of a single quantity.

1 Introduction

Various visual representations, such as error bars, are intended to help the viewer reason about the distribution of values that a random variable could take. For example, examine Figure 1 and try to answer the question posed below the figure (Answer is below[1]). If you find it difficult to answer the question using the plot, you are not alone. Our research finds that over half of the

[1] $Pr(B > A) \approx .75$ (i.e., 75%)

viewers will underestimate the true probability by 0.5 (the majority guessing somewhere around 0.2 or 0.25 as their answer).

The visualization you viewed in Figure 1 presents information about a probability distribution for each variable shown. In doing so, the plot aligns with guidelines for reporting quantitative data that suggest presenting such distributional information whenever observed values may not reliably reflect the underlying population [1]. For example, a weather forecasting model may offer a probability distribution over weather outcomes at a particular time. As another example, consider outcome data from 32 subjects in a lab experiment. From the data, a sampling distribution of the mean is derived. It provides a model of the probability density of the mean outcome for other hypothetical experiments with 32 subjects, assuming the true mean and standard deviation are those estimated using the data from the real subjects. The task of uncertainty depiction is to visually represent the distributional information so that a user can integrate it into their interpretation.

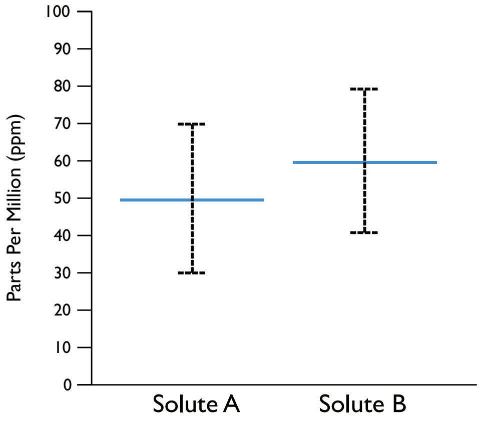

Figure 1. Fictitious scenario. Scientists have measured the concentration of two chemical solutes (A and B, each measured in parts per million) in many vials of sea water. Horizontal blue lines are the means, dashed vertical bars capture 95% of the measurements for each solute, and you may assume independence. Question: in what percentage of vials is there more of solute B than A ($Probability(B > A)$? Answer below.

An error bar =like that in Figure 1 or Figure 2 left provides a static, abstract representation of a univariate distribution. The error bar shows a range around the central tendency (e.g., mean or median). The length of the bar may represent 1 standard deviation, or enough to cover 95% of the random draws from the distribution. When the error bar is based on a sampling distribution of the mean of some underlying distribution, it is called a confidence interval. Unfortunately, despite their widespread usage, the interpretation of errors bars, is notoriously difficult, as evidenced by the high error rate among the viewers we observed of Figure 1. Though our participants are “lay readers” who may not have advanced knowledge of statistics, the high levels of error we observe are unlikely to stem from a lack of expertise. Research shows that even experts fail at making correct statistical inferences from error bars [2].

As alternatives, analysts sometimes use other static, abstract representations of distributions ranging from the well-known box plots to rarer forms such as violin plots (Figure 2, center), gradient plots, and other variants [3]. These forms are often more expressive of the distribution and can avoid biases associated with reading error bars [4]. However, such forms require additional knowledge of the visual encoding to decode the distributional information. They can also require more statistical background knowledge to interpret.

In part, static forms are a necessity of static publication media–i.e., paper. Increasingly, information is presented with electronic formats (e.g., Web pages, PDFs, etc.) augmenting, or even replacing, paper. These new digital forms will increasingly allow us to better convey complex information through features such as interaction and animation. Depictions of distributions, for example, can be dynamic and interactive. Ideally, the new dynamic representations can be coupled with static representations to provide a solution that can work for both electronic and paper transmission of data.

In this paper, we present and study an alternative approach for depicting distributions, which

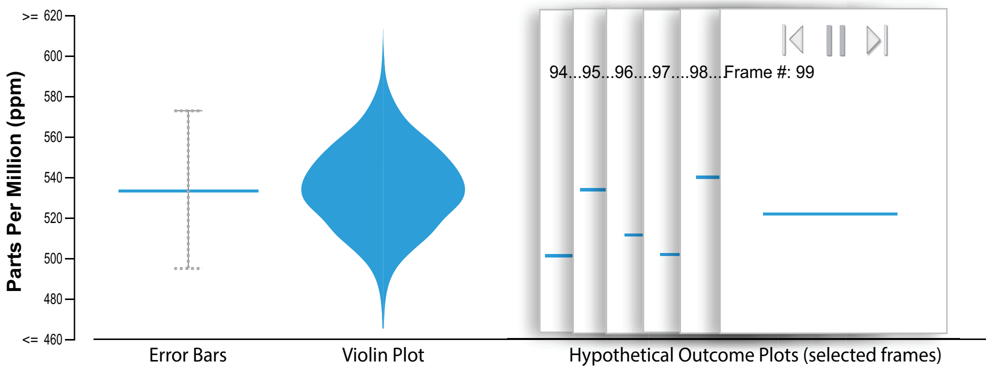

Figure 2. An illustration of our different study conditions. Error bars convey the mean of a distribution of measurements (outcomes) along with a vertical "error bar" capturing a 95% coverage interval. Violin plots extend this idea by showing the distribution in a mirrored histogram. Hypothetical Outcome Plots (HOPs) present the same distribution as animated frames (that can be played in sequence or manually flipped through). Each frame contains a horizontal bar representing one outcome. Click to play the HOPs animation (in Adobe Acrobat).

we call Hypothetical Outcome Plots (HOPs). In its most simple variant, the HOPs approach is to: (1) Draw a sample of hypothetical outcomes (draws) from the distribution; (2) for each, make a plot that becomes one frame in an animated presentation. For example, Figure 2 (right) depicts several frames for a HOPs visualization for a single random variable. Each frame contains a horizontal bar that depicts a specific outcome, one draw for that random variable. An interactive controller allows the viewer to start/pause and step through frames.

There are two clear drawbacks to dynamic presentation of individual draws. First, it introduces sampling error. The reader of the visualization will examine only a finite number of frames, and thus will get an imprecise picture of the complete distribution. We explore this in more detail in Section 6.2. Second, the reader will have to integrate information from multiple frames, either using the visual system or some more mechanical process such as counting. Maintaining visual stability across frames (e.g., by keeping the range of the y-axis fixed) reduces the difficulty of visual integration but does not eliminate it.

On the other hand, there are several advantages to HOPs:

1. HOPs enable viewers to think in *finite terms* (i.e., counts) about *individual outcomes* rather than infinite terms (i.e., probabilities) abstracted over entire distributions, which numerous studies have shown are more difficult for humans to conceive of [5, 6];
2. HOPs do not require an analyst to add new marks (e.g., an error bar) or new encodings (e.g., width, transparency) and do not require viewers to understand those marks and encodings.

We present a study in which subjects make inferences about the probability distributions of one, two, and three random variables at a time using HOPs, error bars, and violin plots. Most critically, our results indicate that HOPs support more accurate inferences about bivariate and trivariate distributions (e.g., the probability that quantity B is larger than A or larger than both A and C). As we might expect error bars and violin plots perform well for simple inferences about univariate distributions. However, and perhaps surprisingly, HOPs achieve comparable performance for many such tasks.

2 Related Work

We review prior work that conveys uncertainty by showing information from probability distributions of one or more random variables, which are either the underlying probability distributions or sampling distributions.

2.1 Static Depiction of Probability Distributions

Many approaches generate a static representation of a probability distribution. A common approach is to add an uncertainty depiction as an extrinsic annotation to a plot. For example, error bars representing confidence intervals can be superimposed on bar charts [7]. Properties of the distribution may also be represented in summary plots using a series of marks (e.g., a boxplot or modified box plot as in [4, 8, 9]). Extrinsic representation can result in interpretation errors, however, because the statistical construct represented by an interval (such as one standard error, or a 95% confidence interval) is not properly understood [2, 9]. Individuals may apply heuristics for reading the error bar that are not correct, such as assuming that overlapping error bars always indicate a non-significant difference [10], or that error bars display a region of uniform probability [9].

Users are also likely to underweight the uncertainty information relative to the underlying data due to the separation of the uncertainty as separate marks. Heuristics based on the "hard" data impact viewers' abilities to accurately integrate information from error bars: viewers who use bar charts with error bars superimposed are influenced by a within-bar bias, perceiving outcomes below the top of the bar as more likely than those above [4, 11].

Other abstract, static representations encode a distribution's probability density function as either marks or "retinal variables" (e.g., color, shape, texture) [12]. For example, violin plots, which we include in our study, encode the probability density as mark width [13–15]. This enables visual inference about the cumulative density function based on the sizes of shaded regions. The gradient plot (Fig. 9) instead encodes density using mark opacity. Several studies that include variants of both find no evidence of a performance difference between the two [4, 9]. We test only the violin plot, as this visualization encodes the probabilities using area, which is considered to be easier to decode than the opacity ~~as is~~ used in the gradient plot [16].

2.2 Depiction of Multiple Individual Outcomes (Draws)

Several previous uncertainty visualizations present multiple individual draws, or possible outcomes from an observed probability distribution rather than abstract representations of distributions. For example, several early presentations used bootstrapping to generate and visualize draws based on observed data for rainfall levels [17, 18]. In more recent geospatial applications, multiple visualizations of spatial models are stochastically generated and presented using random and serial animation and interactive mechanisms such as toggling by the viewer [19–23]. Researchers have proposed that animation is particularly helpful for helping viewers recognize spatial autocorrelation [21]. Evans [22] conducted a comparison between land cover maps that used color saturation to display value certainty levels, maps that displayed only highly certain data, and a "flickering" map that alternated between showing all data and only highly certain data. The flickering maps were found to be helpful overall, though "annoying" to some users. Recently, the New York Times has used simulation-generated samples to illustrate uncertainty in employment projections and potential outcomes of political elections to general audiences [24, 25].

While not animated, hypothesis test "line-ups" and similar approaches to graphical statistical inference also rely on visual comparisons between multiple hypothetical outcomes ([26]; see also [27–30]). In the classic line-up technique, users compare a series of plots. One is based on the real data. The others are drawn from a null hypothesis distribution. If the user can pick out the real data from the "line-up" of plots, then the plausibility of the null hypothesis is

diminished. For example, suppose the dataset is a table with two columns, A and B, each cell containing a numeric value. The plot of the real data would be a scatterplot. Suppose the null hypothesis is that there is no correlation between A and B. Each hypothetical outcome can be generated through permutation bootstrapping. That is, randomly shuffle the values in column B, and make a new scatterplot.

HOPs can be applied more generally to any distribution, including any sampling distribution, not just the null hypothesis distribution. This enables applying HOPs to facilitate statistical inferences beyond Null Hypothesis Significance Testing (NHST). For example, compute the correlation between A and B in the real data and use it to create a model of the joint distribution of A and B. Then, draw samples of the same size from the modeled joint distribution and make scatterplots of them. This would provide the user with a sense of the range of correlations that would be likely to be observed in samples of that size, assuming that the true correlation is the correlation observed in the sample.

Within statistics education, simulation has been used to convey fundamental concepts like sampling distributions and confidence intervals [31]. The motivation is that simulating individual outcomes provides a more concrete way to think about the abstract notion of distributions. Our exploration of HOPs shares the same motivation. However, rather than being a stepping stone for teaching people how to interpret abstract static representations, we suggest that in many situations animated HOPs may be a good substitute for those abstract static representations.

In attempting to reform statistics away from NHST, Cumming suggests the value of the cognitive evidence that is provided by the “dance of the means” and other sample-based simulations for supporting more intuitive understanding of variance, sampling, and related concepts [32]. Dance of the means is animated HOPs with hypothetical outcomes drawn from the sampling distribution of the mean.

No previous work that we are aware of has directly tested the efficacy of dynamic depiction of individual outcomes as alternatives to standard representations, what we are calling animated HOPs. Our primary contribution is to provide empirical evidence that untrained users can interpret and benefit from animated HOPs.

3 Study: Methods

We conducted a user study on Amazon Mechanical Turk to assess people’s ability to interpret HOPs, error bars and violin plots. Each subject was assigned to one of the three representation conditions and was presented with a sequence of nine tasks.

We asked subjects to report numerical properties of the distribution of possible outcomes that HOPs, error bars and violin plots all convey. For example, for a plot representing a single random variable with a continuous outcome, we asked people to report the mean of the distribution, the probability of an outcome above some threshold k_1, and the probability of an outcome between two other thresholds, k_2 and k_3. The outcome of interest is the absolute error, the absolute value of the difference between the subject’s report and the correct answer. We conclude that one technique is better than another if subjects tend to have smaller absolute errors in the answers they give.

Some attempts to measure the effectiveness of uncertainty representations have asked people to view a representation, report some property, and then express how confident they are about the report. We find this approach problematic because there is no correct amount of confidence to report. In such experiments, there is no way to assess the correctness of responses about individual plots. It is only possible to infer that people are reading multiple plots inconsistently: for example, if subjects express more confidence about an outcome in plot 1 than plot 2, but the outcome distribution in plot 1 is actually more dispersed than the one in plot 2, then there must be something wrong with the representation of uncertainty in the two plots. For this reason, we chose to use the approach in the canonical work of Ibrekk and Morgan [9], who presented

various representations of uncertainty for weather forecasts and asked subjects to report probabilities (e.g., snowfall > 2 inches, or between 2 and 12 inches).

3.1 Apparatus

Values are drawn from normal (Gaussian) distributions. In the simplest plots, there is just one variable, A. We also present plots of two variables, A and B, and of three variables, A, B, and C.

The most common use of error bars is to present confidence intervals depicting information about a sampling distribution. For example, suppose we have a set of 32 vials of seawater and have measurements of the number of parts-per-million of a chemical solute in each vial. We imagine drawing many other sets of 32 vials from the same underlying distribution. The distribution of the means of those samples is the sampling distribution.

By contrast, for our tasks we framed the inferences to be made as reading off properties of an underlying distribution rather than a sampling distribution. This kept the task descriptions simple. It also reduced opportunities for misconceptions about statistical inference from affecting people's answers to the questions. The instructions explain:

> Scientists have measured the concentration of some chemical solutes (measured in parts-per-million) in many samples of sea water. We will show you plots based on their measurements and ask you questions about them.

Thus, the error bars that we present are not confidence intervals. They do not show a property of the sampling distribution of means of many vials. Instead, they show a range of values that cover 95% of the underlying distribution.

The text explaining the error bars reads:

> The blue line shows the average amount of solute in all the seawater vials. The dashed lines show an error bar, a range above and below the average. 95% of the collected vials fall in the range defined by the dashed lines.

Similarly, the violin plots also show the probability density function for the underlying distribution, not a sampling distribution. The text explaining the violin plots reads:

> The width of the colored area at each level shows how many vials of sea water were found to have that particular amount of the chemical solute.

The text explaining the animated HOPs explains that each frame shows one draw and also provides instructions for manually controlling the animation. It reads:

> Each plot shows the quantity of solute in one vial of seawater. Use the buttons at the top of the plot to pause, play, or step forward and back through the plots if you want to see them at your own pace.

To encourage subjects to read these descriptions, for the first trial each subject must click a button at the top of the plot to reveal the questions (and start the animation for HOPs). After the subject submits answers to the questions for the first distribution, the second plot is shown. The questions display immediately and in the HOPs condition the animation starts immediately.

In the HOPs condition, the animation advances every 400ms. Subjects could pause the animation and move forward and back one frame at a time. We note that 400ms provides enough time for eye motion and silent-counting [33]. The animation looped after the 5000 frames had completed, which was clearly conveyed to the user by restarting the frame numbering at 0.

All visualization stimuli were created using D3 [34]. The visualization software and study interface are available in Supplemental Material.

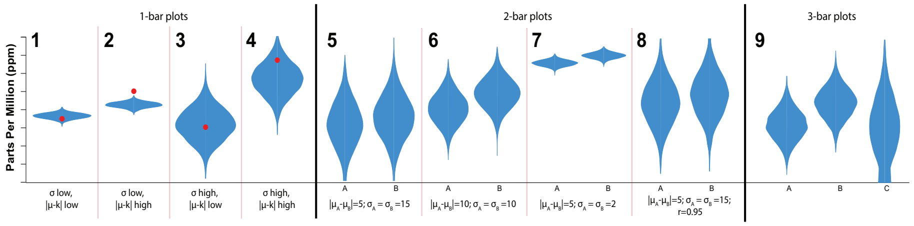

Figure 3. Violin plots showing the four one-variable distribution types, the four two-variable distributions, and the single three-variable distribution summarized in Tables 1 - 3. Click the image to play the HOPs animation.

Table 1. One-Variable Distribution Types

Distr.	σ	$D = \|\mu - k_1\|$	$Pr(X > \mu + D)$
1	3	5	5%
2	3	20	0%
3	17	5	39%
4	17	20	12%

Table 2. Two-Variable Distribution Types

Distr.	μ_a	σ_a	μ_b	σ_b	$corr(A,B)$	$Pr(B > A)$
5	40	15	45	15	0	59%
6	50	10	60	10	0	76%
7	80	2	85	2	0	96%
8	55	15	60	15	0.95	85%

Table 3. Three-Variable Distribution Types

Type	μ_a	σ_a	μ_b	σ_b	μ_c	σ_c	$Pr(B > A \text{ and } B > C)$
9	40	10	55	10	40	20	66%

3.2 One-Variable Tasks and Distributions

Each subject completes four trials, each with one univariate distribution. For each trial, the subject reports three properties of the distribution, the mean and two properties of the Cumulative Distribution Function (CDF). Because previous research has shown that people are better able to reason about probabilities when they are expressed as frequencies [5,6], we ask subjects to report frequencies as x times out 100.

1. What is the average measurement of solute in parts-per-million (ppm)?
2. How often are the measurements above the value of the red dot?
3. How often will the measurements lie between k_2 and k_3 ppm?

Each trial is defined by the parameters: mean (μ), standard-deviation (σ), distance of the red dot from the mean (k_1) and whether the red dot was above or below the mean. We always set k_2 to be the closest multiple of 10 below below μ and k_3 to be closest multiple of 10 that is at least 20 above μ. We deliberately varied these parameters between trials and subjects to reduce the possibility of results that are limited to particular parameter sets.

In particular, σ was either *low* (3) or *high* (17), and the distance between μ and the red dot either *small* ($\pm$5) or *large* ($\pm$20). We randomize whether the red dot is k_1 units above or below μ. Table 1 summarizes the four distribution types. The last column shows the probability that the value will be higher than the red dot when the red dot is above μ. When the red dot is below μ, the correct probability is the inverse of that shown (e.g., 88% instead of 12% for distribution type 4).

To ensure that there is nothing special about the particular μ values, and to ensure that subjects don't think of additional tasks as related to previous ones, for each distribution type we construct four distributions with different values of μ: 131, 344, 523, and 672. We always set k_2 to be the closest multiple of 10 below below μ and k_3 to be closest multiple of 10 that is at least 20 above μ

Each subject completes one trial for each of the four distribution types in Table 1. To counter-balance for any possible order effects, we construct all 24 possible orderings of the four distribution types. Each of those is crossed with four orderings of the μ values, generated from a 4×4 Latin Square to assure that each μ value appears equally often as the first, second, third, or fourth trial. That yields $24 \times 4 = 96$ possible sequences of four trials. For each sequence, we randomly ($Pr = 0.5$) determine whether the red dot is above or below μ for each of the four distributions. The 96 possible sequences are fixed. For each of the visualization conditions (HOPs, violin plots, and error bars), one subject is assigned to each sequence of four trials.

For each distribution in each sequence, we simulate 5000 draws from a normal distribution. This same set of 5000 draws is used to generate all three visualizations for that distribution. Some drawn values are below 0. Since negative values do not make sense as amounts of a chemical solute, all such values are rounded up to 0 regardless of visualization condition (we repeat this step for two and three variable distributions below). All violin plots are generated in D3 [35] using the histogram function with fine-grained bins. Figure 3 shows violin plots for all the distributions.

3.3 Two-Variable Task and Distributions

After completing all four one-variable trials, each subject completes four trials involving bivariate distributions. For each trial, each subject reports a single property of the joint distribution: how often is the value of the random variable B larger than that of A. The exact wording was:

> How often is the measurement of solute B larger than the measurement of solute A? Answer in terms of the number of times out of 100.

A common inference that people are expected to make from a pair of error bars is the statistical significance of a difference in population means. In that case, the error bars display confidence intervals around sample means, an interval that covers 95% of sampling distribution of the mean. For example, how plausible is the null hypothesis that the average heights of women and men are the same given the mean heights and variability found in samples of n women and n men? This involves some visual inference: assessing whether $Pr(B > A)$ is above some threshold, where A and B are sampling distributions of the means. It also involves some statistical inference: if, $Pr(B > A)$ is above some threshold, then the difference in means is statistically significant.

Our experiment does not ask subjects to judge the statistical significance of a difference in means, for two reasons. First, judgments of significance require both visual and statistical inference. Prior work has shown that people often get confused by the statistical inference associated with sampling distributions [2]. We therefore chose to isolate the visual inference task: the ability to decode the visualization to make probability estimates. Thus, we do not present sampling distributions at all and our error bars convey coverage rather than confidence intervals. We ask subjects to estimate the percentage of vials where B is larger than A. We do not refer to samples of many vials.

Second, we wanted to get a finer-grained measure of people's ability to assess the reliability of A>B. Thus, rather than asking a binary question of reliability (is $Pr(B > A)$ above some threshold such as 95%), we asked directly for $Pr(B > A)$. If there is special interest in knowing whether people make qualitative errors, such as thinking $Pr(B > A) > .95$ when it is not we can set various qualitative thresholds such as .95 during the analysis phase and count the frequency of errors rather than their average magnitude.

We deliberately vary the means and standard deviations between A and B to generate tasks where $Pr(B > A)$ varied. The parameters for each of three data sets are shown in Table 2, along with the correct value of $Pr(B > A)$ that subjects were supposed to report. In distributions 5-7 in Table 2A and B are independent. Distribution 8 is identical to distribution 5 except that the

means are shifted up and the correlation between A and B is very high. The correlation causes $Pr(B > A)$ to be high despite the relatively small difference in means.

To counter-balance for any possible order effects, we construct all 24 possible orderings of the four distributions. In each of three conditions (HOPs, error bars and violin plots) we assign four subjects to each of the possible orderings. For each task, we simulate 5000 draws from the joint distribution defined by the appropriate row in Table 2. This same set of 5000 draws is used to generate all three visualizations for that data set.

3.4 Three-Variable Task and Distribution

After completing four two-variable trials, each subject answers one question about a trivariate distribution. Subjects are asked to judge how often value B is the largest of the three. The exact wording was:

> How often is the measurement of solute B larger than both the measurement of solute A and the measurement of solute C? Answer in terms of the number of times out of 100.

The three random variables are independent, defined by the parameters in Table 3. We again simulate 5000 draws. The same set of 5000 draws is used to generate all three visualizations for that task. Note that for value C in the trivariate violin plots in Figure 3, there is a bulge at zero reflecting the negative numbers rounded up to 0 in that distribution.

3.5 Subjects

The number of subjects is based on a prospective power analysis. The experiment was powered based on the bivariate distribution questions, where subjects have to estimate the probability that $B > A$. We use pilot data from a previous experiment using slightly different versions of error bars and HOPs and a different framing of the task. From the previous experiment, we get standard deviations in the mean absolute error of subjects' estimates of 11 for HOPs and 18 for error bars.

We power the study to detect a true difference in mean absolute error of ten percentage points between two visualization conditions. Through simulations, we find that with 90 subjects per treatment, the null hypothesis will be rejected 82% of the time when the true difference in error rates is 10%. Some of the counter-balancing of potential order effects was easier with a multiple of 24 subjects, so we actually run 96 subjects per condition, for a total of 288.

Subjects were recruited on Amazon Mechanical Turk (AMT). AMT has been shown to be an effective platform for conducting graphical perception studies [36], and has been used in the information visualization community for testing how non-statisticians interpret visualizations of uncertainty [4]. The population of workers is largely gender balanced, and consists primarily of individuals in their 30's and 40's with median household incomes similar to that of the U.S. median [37]. We used a standard selection criteria that restricted the task to U.S. workers with an approval rating of 95% or above. Subjects received a base reward of \$0.90 and a bonus of \$0.15 times the total number of distributions, or trials (out of nine total) for which they correctly answered the question (one of their three responses for each univariate distribution trial was randomly drawn and scored for the bonus). Hence, the maximum payment a subject could receive was \$0.90 + \$1.35. The HIT duration was limited to 30 minutes. No other time restrictions were applied. As a quality control measure in analysis, on each of the univariate distribution trials subjects were asked the value corresponding to the red dot. The range of the y-axis was always 160. Subjects who were off by more than 30 on the value corresponding to the red dot for any of the four trials were deemed deficient in either effort or ability to make inferences from an x-y plot. One subject in the HOPs condition, 4 in the violin plot condition, and 7 in the error bars condition were deficient in this way. Their data was discarded and each

was replaced by a new subject completing the same task sequence. We recorded the total number of subjects who started the experiment but did not complete all the tasks. The dropout rate was 13.1% for HOPs, 14.4% for error bars, and for 9.4% for violin plots. Subjects who dropped out were also replaced with new subjects. All of the analysis is reported based on the final 288 subjects who completed all nine trials and passed the quality control check.

3.5.1 Ethics

The study was approved by the University of Michigan Health Sciences and Behavioral Sciences Institutional Review Board (Study Number HUM00065618). Subjects were presented with study information and terms of consent on the introductory task screen and expressed their consent by clicking 'Continue' to start the task.

4 Hypotheses

We expect that subjects will be able to estimate the mean of the distribution very easily in the error bar condition as the mean is shown explicitly and the description below the plot states which line represents the mean. We also expect that subjects will be able to estimate the mean of the distribution easily with a violin plot: since our distributions are normal and contain enough samples, the mean is also the median and the point of highest density (the widest part of the violin plot). For these plots, any errors should come only from misunderstanding the nature of the plots or from imprecision in visually tracking from a point on the graph to a point on the y-axis and interpolating an exact value between labeled tick-marks on the y-axis.

Estimating the mean from animated HOPs is not quite so easy. The subject has to view multiple frames and integrate information across them to estimate an average vertical position. There is an additional source of imprecision introduced by looking at only a finite set of frames: even if the subject perfectly interprets n frames, the standard error when estimating the mean will be $\sigma/\sqrt{n}$. When σ is small, then, subjects should estimate the mean quite well, even when viewing only a few frames. Indeed, with just one frame, the precision will be σ. When σ is large, however, it is harder to integrate the information across frames (the lines jumps around a lot between frames) and more frames are needed to get a precise estimate. Thus, we have:

Hypothesis 1. *When estimating the mean of a single variable, subjects will have lower error rates using error bars and violin plots than HOPs where σ is high (distributions 3 and 4), but not on distributions where σ is low (distributions 1 and 2).*

In principle, violin plots are an excellent representation for allowing inferences about the cumulative density above certain thresholds or between two thresholds. A subject just needs to estimate what portion of the total shaded area falls in the desired range. Error bars are less suited to this task. A subject who is very familiar with the normal distribution may be able to mentally visualize the cumulative density function from the mean and the 95% interval, or may know some analytic properties of the distribution (e.g., about 32% of values are more than σ away from the mean), and then make further inferences to yield an estimate. There is not, however, a simple visual procedure that will yield accurate estimates. With animated HOPs, subjects have to integrate visually over many frames or count occurrences above the threshold. In addition, there will be imprecision from examining a finite number of frames. Thus we have:

Hypothesis 2. *When estimating the probability of a random variable above a threshold or between two thresholds, subjects will have lower error rates using violin plots than error bars or HOPs.*

It is more difficult to assess the reliability of a comparison between the joint distribution of two random variables using error bars or violin plots. For distributions 5-7, where the two random variables are independent and normally distributed, both error bars and violin plots in

Table 4. Mean Absolute Error by Treatment and Task Parameters. The $*$ indicates a significant difference between the starred treatment and at least one other ($p_{adj} < 0.001$). Figure 3 illustrates the violin plot for each Type.

	1-var					Mean Absolute Error		
Row	Type	σ	$\lvert\mu - k_1\rvert$	Question	Correct Answer	HOPs	Violin	Err. Bar
1	**1,2**	low	N/A	μ_A		3.7	3.8	7.8
2	**3,4**	high				9.6*	4.2	2.1
3	**1**	low	low	$Pr(A > k_1)$	.05	0.14	0.35*	0.21
4	**2**	low	high		0	0.06	0.06	0.09
5	**3**	high	low		.39	0.13	0.10	0.11
6	**4**	high	high		.12	0.14	0.11	0.14
7	**1,2**	low	N/A	$Pr(k_2 \leq A \leq k_3)$		0.20	0.19	0.29*
8	**3,4**	high				0.17*	0.14	0.13
	2-var	$\mu_a - \mu_b$	$\sigma_a = \sigma_b$					
9	**5**	5	15	$Pr(B > A)$	.59	0.11*	0.37	0.38
10	**6**	10	10		.76	0.13*	0.35	0.42
11	**7**	5	2		.96	0.09*	0.45	0.54
12	**8**	5	15		.85	0.10*	0.63	0.60
	3-var							
13	**9**			$Pr(B > A \text{ and } B > C)$	.66	0.14*	0.37	0.36

principle provide complete information about the joint distribution. However, there is no simple visual operation that yields the correct answer. In addition, for distribution 8, neither error bars nor violin plots convey information about the correlation between A and B while HOPs do. HOPs, on the other hand, are still straightforward to interpret. Indeed, it may be easier visually to assess whether $B > A$ in one frame than to assess whether a draw from a univariate distribution falls in some range.

Thus, we have:

Hypothesis 3. *When estimating $Pr(B > A)$ in a bivariate distribution plot, subjects will have lower error rates using HOPs than violin plots or error bars.*

For the task of estimating how often B will be the largest of three random variables, error bars and violin plots again provide complete information about the joint distribution, but in a format that does not afford easy visual inference. By contrast, we expect that subjects using HOPs will do almost as well on the three-variable trial as the two-variable trials. Thus, we have:

Hypothesis 4. *When estimating $Pr(B > A$ and $B > C)$ in a trivariate distribution plot, subjects will have lower error rates using HOPs than violin plots or error bars.*

5 Study Results

5.1 Preliminary Steps

Analyzing the total time to completion of the 288 subjects showed that HOPs viewers spent an average of 523s (median: 544s) to complete the entire study, slightly longer than error bar and violin plot viewers (means: 479s, 481s, medians: 451s, 451s, respectively) but the difference was not statistically significant ($F(2,285) = 2.02$, $p > 0.1$).

Many HOPs subjects (35%) used the interactive features (pause and stepping forward or backward through frames) on the first question screen. However, on average, just 10% of subjects used these features on each of the subsequent screens, varying only slightly between the one-, two-, and three-variable tasks (9.7%, 9.8%, and 13.5% respectively). The median number of frames displayed, over all screens (data sets), was 89 (mean: 101, SD: 79). We observed no significant effects on error rates in subjects' responses from the number of frames, number of interactions, or time spent on page prior to answering each question.

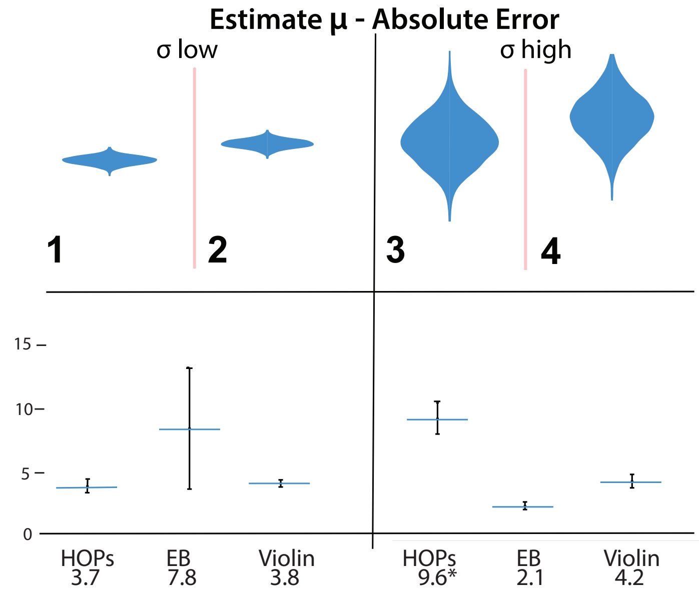

Figure 4. Stimuli (top) and absolute error (bottom) of estimates of μ. Error bars in the results plot show a 95% confidence interval. Click the image to play the HOPs animation. Each HOPs frame is a draw from the sampling distribution.

Results for the one-, two-, and three-variable trials reported below include all 288 subjects. We ran all models reported below with and without an indicator variable for the order in which the subject completed the trial and a variable that captured the time the subject spent on the page. We saw no main effect of either variable in any analysis, and improvements in R^2 of 1% or less from including order and time on page. All models below therefore omit order and time on page.

5.2 One Variable Results

5.2.1 Estimating μ

For the question that asked subjects to estimate the mean, we hypothesized that high variance data would make the inference more difficult for HOPs viewers, while low variance data would yield no differences in the accuracy of mean estimates between treatments (H1). Our results support H1: we observe a significant difference between mean absolute error between treatments for the high variance dataset ($F(2,573) = 32$, $p < 0.001$, Table 4 row 2) and no difference in MAE between treatments for the low variance data ($F(2,573) = 0.9$, $p = 0.4$, row 1). Specifically, for the high variance data set we find that the HOPs viewers show significantly higher levels of error compared to both error bars and violin plots (both $p_{adj} < 0.001$). While no significant difference exists for the low variance data set, the mean absolute error for the error bars is noticeably higher than that of the other two conditions. Deeper examination of the data revealed an outlier with very high error for estimating μ for one low variance dataset. If we remove the outlier, we see a slightly lower mean absolute error for error bar users (1.9) which is significantly lower than both other conditions ($F(2,571) = 10.7$, $p < 0.001$, both $p_{adj} < 0.001$).

5.2.2 Estimating $Pr(A > k)$ and $Pr(k2 \leq A \leq k3)$

We hypothesized that subjects would be more accurate in estimating the probability that A is greater than k_1 or between the values k_2 and k_3 with violin plots given that these plots directly depict information from the pdf as area (H2). Our results provide little support for H2. On one

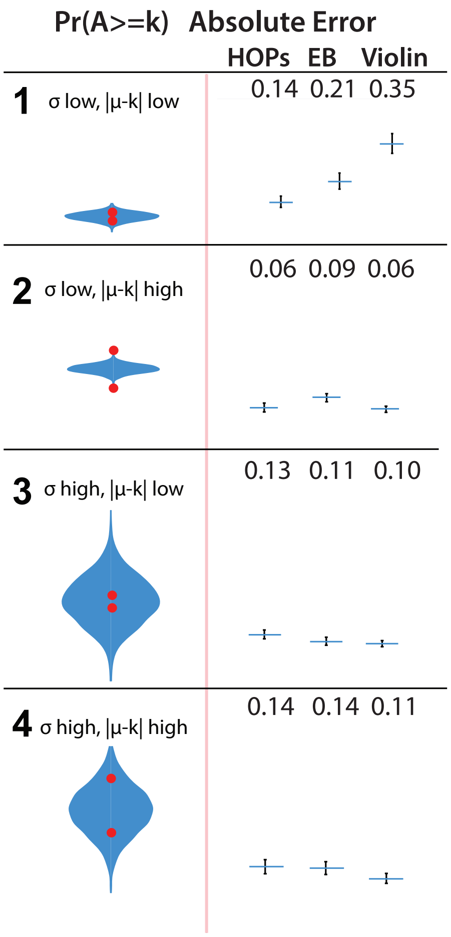

Figure 5. Stimuli (left) and absolute error (right) of estimates of $Pr(A \geq k)$. Both locations of the red dot (above and below $_mu$) are shown, though subjects saw only one of the dots in each trial. Error bars in the results plot indicate a 95% confidence interval. Click the image to play the HOPs animation. Each HOPs frame is a draw from the sampling distribution.

task of estimating $Pr(A > k)$ was MAE lower for violin plots than either of the other two. In row 3, where σ is low and $|\mu - k_1|$ is low, we see significantly higher MAE among viewers of the violin plots.

In row 7, error bars performed significantly worse than violin plots and HOPs ($F(2,573) = 15$, $p < 0.001$, both $p_{adj} < 0.001$). In row 8, HOPs performed significantly worse than violin plots and error bars ($F(2,573) = 15$, $p < 0.01$, $p_{adj} < 0.05$ and $p_{adj} < 0.01$ respectively).

5.3 Two Variable Results

We hypothesized that subjects who used HOPs for two-variable plots would make more accurate inferences about how often $B > A$ (H3). Our results provide strong support for H3. For all three two variable plots, subjects who used HOPs had much lower MAE than those used violin plots and error bars ($F(2,573) = 73, 57, 84, 220$, all $<= 0.001$, all $p_{adj} < 0.001$, rows 9 through 11 respectively).

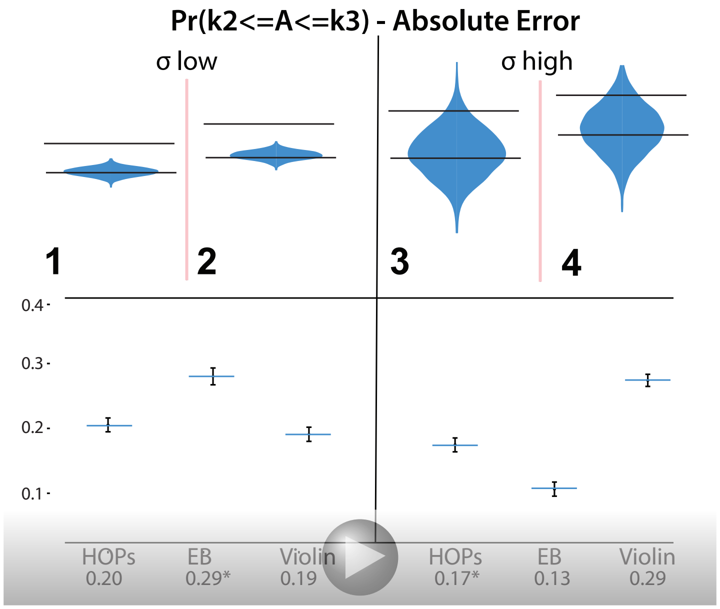

Figure 6. Stimuli (top) and absolute error (bottom) of estimates of $Pr(k_2 <= A <= k_3)$. Error bars in the results plot indicate a 95% confidence interval. Click the image to play the HOPs animation. Each HOPs frame is a draw from the sampling distribution.

5.4 Three Variable Results

We hypothesized that subjects who used HOPs would make more accurate inferences about how often B was the largest of the three values (H4). Our results provide strong support for H4. Subjects who used HOPs had lower MAE than those who used violin plots and error bars ($F(2,573) = 43$, $p < 0.001$, both $p_{adj} < 0.001$, row 13).

6 Discussion

The experiment tasks were about as favorable as possible for the abstract, static representations. Normal distributions lead to symmetric violin plots where the widest point is the mean and median. To the extent that people have developed intuitions about error bars, they will have developed them for independent, normal distributions. Even under these most favorable conditions, performance with animated HOPS was comparable on the one-variable trials and much better on the two- and three-variable trials.

Animated HOPs performed worse on some types of one-variable trials. One was estimating the mean when the variance was high. As we argued in the Section 4, this was expected for two reasons. First, the large variance meant subjects had to visually integrate over large distances when the line jumped around a lot. Second, the imprecision due to examining a finite number of frames was higher when the variance was larger. HOPs users were also slightly worse at estimating the cumulative density of values between two thresholds when the variance was high.

HOPS also performed better on some one-variable trials, in particular estimating cumulative densities when the variance was low. Violin plots had higher errors when estimating the probability of a value above a threshold. Error bars had higher errors when estimate the probability of values between two thresholds. We speculate that the low variance meant the violin plots and error bars were compressed vertically, and thus it was difficult to assess exactly where in the distribution a particular level fell, and how much area was above or below it. It is not clear, however, why violin plots were especially problematic for estimating $Pr(A > k_1)$

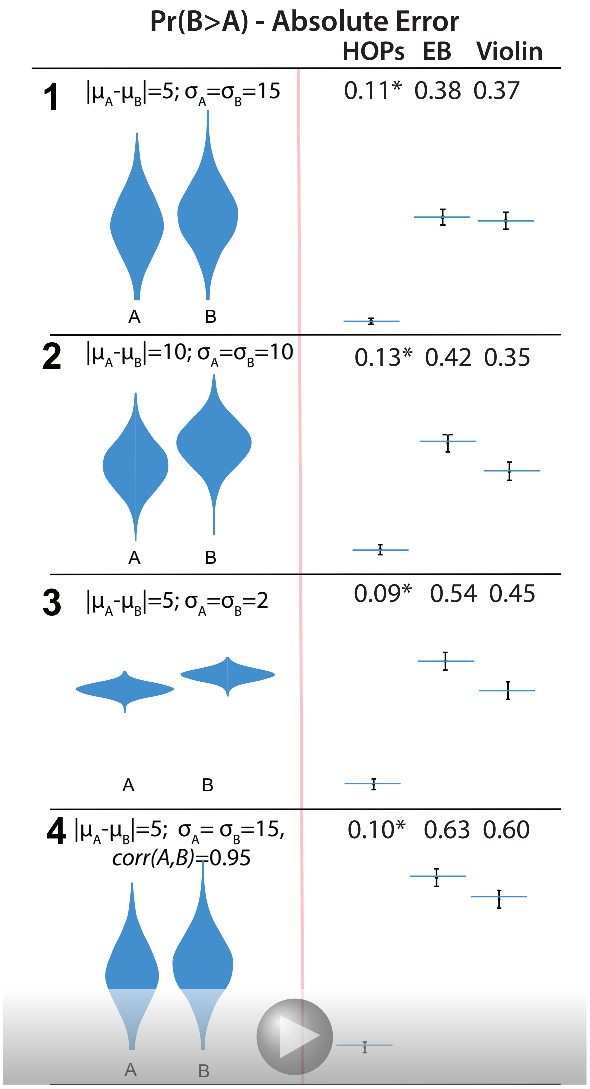

Figure 7. Stimuli (left) and absolute error (right) of estimates of $Pr(B > A)$. Error bars in the results plot indicate a 95% confidence interval. Click the image to play the HOPs animation. Each HOPs frame is a draw from the sampling distribution.

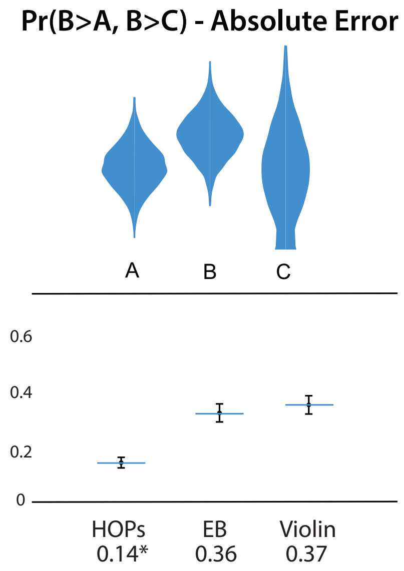

Figure 8. Stimuli (top) and absolute error (bottom) of estimates of $Pr(B > A, B > C)$. Error bars in the results plot indicate a 95% confidence interval. Click the image to play the HOPs animation. Each HOPs frame is a draw from the sampling distribution.

while error bars were especially problematic for estimating $Pr(k_2 \leq A \leq k_3)$. Additional research is needed to assess the robustness of this finding and develop an explanation for it.

In light of the common usage of error bars for presenting multiple independent distributions, it is noteworthy how poorly subjects using these representations performed on tasks asking them to assess how reliably a draw from one variable was larger than the other(s). On no task was the mean absolute error less than 36 percentage points. That means, for example, that on row 9 (task type 5), where the correct answer was 59%, subjects gave mean answers centered around 95% and 23%(!) Many subjects reported values less than 50%, which are not plausible given that the mean of B was larger than the mean of A. Performance was so poor with the abstract, static representations that we suspected something must have gone wrong with our instructions or recording of results. After careful checking, however, we did not find any obvious patterns to the errors. We speculate that many subjects simply had no idea how to make a good guess. Some may have followed the heuristic of mentally substituting a simpler question, such as the mean difference between B and A. Subjects from the same pool who used HOPs, on the other hand, showed markedly better performance.

A question that arises from our results is exactly what strategies people use in order to infer properties of the distribution when interacting with HOPs. In informal pilots, different people appeared to employ different heuristics in integrating over multiple frames. Some explicitly performed silent-counting [33] whereas others generated estimates in some other way. One possibility is that viewers are making use of ensemble representations which allow for a form of "averaging" across multiple objects and which have been demonstrated to facilitate statistical reasoning [38]. Recent work has demonstrated that these mechanisms can also be employed in temporally-varying situations [39] similar to HOPs. It is also possible that different heuristics are used in different situations or combined in some way. Our own experiences with using HOPs lead us to speculate that people may be combining counting of outcomes that display a pattern of interest and approximation. For example, a viewer might count the number of frames in which B is the largest in our three-variable task for a short while, then estimate the total number of frames they viewed while counting to infer the probability. Further work is necessary to determine if one strategy is better than another in producing estimates.

We expect HOPs to be better for estimating very reliable outcomes regardless of the strategy a viewer employs. If an outcome almost never occurs, it is easy to notice and count its occurrences, even with a fast frame rate. If an outcome almost always occurs, it is easy to notice or count when it does not. For intermediate frequencies, however, it may be more difficult, and performance is more likely to be impacted by frame rate. The results of our study do not provide clear evidence on this conjecture. In row 4, where $Pr(A > k_1)$ approaches 1, the MAE for HOPs was lowest. On the other hand, the next best MAE for HOPs was row 11, where $Pr(B > A)$ was 76%, an intermediate level of reliability.

6.1 Limitations

We note several limitations of our study. First, we know relatively little about our Mechanical Turk subject pool. We followed common practices for ensuring high-quality participation, such as restricting participation to U.S. subjects, paying a relatively high wage, informing subjects of incentive bonuses for correct answers, and discarding subjects who incorrectly answered questions about the location of the red dot. The subjects were probably above-average in numeracy relative to the entire U.S. population, since they understood error bars and violin plots well enough to have low error rates on some of the one-variable trials. We do not know, however, whether specific sub-populations may perform better or worse with HOPs or with the abstract, static representations. An important step for future work is to perform a similar experiment with other subject pools, such as individuals who have recently completed a course in probability and statistics and individuals who have taken such a course some time in the past but not recently.

Second, the tasks we employed may have been special in some way. For example, while subjects could do reasonably well at estimating the mean and cumulative densities of a single random variable using HOPs, we did not ask them to perform those same inferences for one of the variables presented in a two- or three-variable animation. It might be difficult to tune out the bar for another variable and focus only on one variable at a time. It also might be difficult to perform pairwise comparisons of variables that are visually separated, such as assessing $Pr(A > C)$ in a three-variable plot where B is shown between A and C.

Third, our study described the plots as showing values observed in actual samples. In practice, HOPs may be used to convey outcomes generated from a single input data set, such as the mean of the data. The framing of the individual HOPs would then need to describe the idea of a hypothetical outcome as another draw from the sampling distribution. An important next step is to replicate our results with summary statistics that describe the sampling distribution, such as error bars that instead represent a 95% confidence interval. For a lay audience, of course, this would need to be done without reference to sampling distributions. It is not clear whether it is possible to do this effectively.

Fourth, we did not test all abstract, static representations. There are many other possibilities besides error bars and violin plots. Some of them may have better performance.

Fifth, we did not test special purpose representations that were tuned to the particular comparative questions that we asked. For example, in the two-variable case we could have created the composite variable $B - A$ and shown error bars or violin plots for its distribution. For the three-variable case we could have have created the composite variable $B - max(A, C)$ and shown a representation of its distribution. For any specific analytic question, of course, the representation that makes it easiest to answer that question is simply to show the answer. Generally, the goal of a visual representation is to provide intuitions and a chance to explore for patterns. In order to assess whether subjects had developed a good intuitive sense of the data that was presented, we asked specific analytic questions. Tailoring the visual representation to those specific questions would, in some sense, be cheating. Thus, we considered only generic representations of distributions that can support estimation of answers to many analytic questions.

Sixth, we did not test any static representations of finite (concrete) outcomes. One intriguing

possibility are one-dimensional scatterplots of hypothetical outcomes, or variants such as the beeswarm plot [40]. These would have the advantage of not requiring users to integrate visually over time. Untrained users, however, may find it easier to understand each frame in an animation as a representation of one concrete outcome than to understand each dot in a static representation as a representation of one concrete outcome. Further research is needed.

Finally, we did not prompt subjects to explain their conclusions about the data or uncertainty. Subjects' explanations of how they thought about answering the questions might have provided clues about whether they were explicitly counting with HOPS and what heuristics they were using to answer the questions with the two- and three-variable error bar and violin plots.

6.2 Precision of Inference from HOPs

How many hypothetical outcomes from a distribution does a viewer need to see in order to assess properties of the distribution like the mean, the standard deviation, or the probability that the value will be above a threshold k? This number will be impacted by the distribution of the target property, as well as how effectively the viewer processes and integrates the information. We can, however, establish an upper bound on the precision of inferences if we assume that the viewer perfectly processes the information presented.

For example, suppose that the viewer's task is to give an estimate $\overline{\mu}$ of the mean μ of a continuous distribution X with standard deviation σ. The viewer is presented n frames of an animated slideshow, where each frame presents one real number outcome drawn from the distribution. This is the classic task that introductory statistics textbooks use to introduce the idea of sampling distributions. The best estimate of the mean of the distribution is the mean of the n hypothetical outcomes (a sample of size n). The precision of that estimate is inferred from properties of the sampling distribution of the mean. That is, we imagine taking infinitely many samples from the original distribution, each sample being of size n, and collecting the means of all those samples. The collected means come from a distribution which is called the sampling distribution of the mean. According to the Central Limit Theorem, as n gets large, the sampling distribution of the mean will be normally distributed, even if the original distribution was not normally distributed. The standard deviation of the sampling distribution of the mean will be $\sigma_{\overline{X}} = \sigma/\sqrt{n}$. Thus, $\sigma/\sqrt{n}$ expresses the precision that will be possible when estimating the mean of X based on n samples. If $\sigma = 10$ and $n = 25$, then 95% of samples will have estimates for $\overline{\mu}$ within $1.96 * 10/\sqrt{25} = 3.92$ of the true μ. Looking at more frames in the HOPs animation will increase the potential precision, giving a smaller confidence interval, but the value of extra frames diminishes. With four times as many frames, 100 rather than 25, the size of the confidence interval is cut in half.

As another example, suppose that the viewer's task is to give an estimate of $p = Pr(X > k)$. One possible procedure would be to notice, for each frame whether the displayed value is bigger than k or not, without noticing how much bigger or smaller. Then, the subject could count the number of frames out of n where $X > k$ and use that to estimate the true probability p. Again, it is possible to derive analytically the properties of that sampling distribution. The standard deviation of the sampling distribution will be $\sqrt{p(1-p)/n}$. Thus, for example, if $p = .66$ and $n = 25$, the standard deviation will be 0.094, meaning that 95% of the time the estimated probability $\overline{p}$ will be within ± 19 percentage points of the true mean p. Looking at 100 rather than 25 HOP frames would cut this in half; 95% of the time the estimated probability will be within ± 9 percentage points of the true mean.

Of course, viewers are not restricted to coding each frame in a binary way, as the presence or absence of some property. For the task of assessing $p = Pr(X > k)$, the viewer may intuit something from how far particular values are above or below k. Thus, they may be able to make more precise estimates than ± 9 points after viewing 100 frames. However, as the outcome plots and tasks get more complicated, it may become harder to do more with the plot than just noticing whether a binary property is true.

6.3 Frame Rates and Interactivity

The frame rate, how quickly the animation advances to the next frame, is another important design consideration for animated HOPs. Faster frame rates are likely to make it easier to perceive trends such as the variability of a value between frames. It is also likely that a viewer will see more frames with a faster rate than a slower rate, increasing the precision of their estimates. On the other hand, faster frame rates will also make it more difficult to infer properties of each frame.

Deriving the optimal frame rate and understanding how it relates to different types of inferences and properties of the data distributions is an important area for future exploration of HOPs. We use a frame rate of 400ms for our comparative experiment between HOPs and static alternatives. Viewers in pilot studies identified this rate as effective for the target inferences in our study (estimating $Pr(A > k)$, etc.) without being too fast (resulting in reports from viewers of mental fatigue) or too slow (resulting in reports that integrating information across frames was too difficult). We note that 400ms provides enough time for eye motion and silent-counting [33]. As noted above, the accuracy of inferences for very reliable outcomes are less likely to depend on the frame rate. Viewers may prefer faster frame rates than 400ms in these cases for more efficient use of HOPs.

Because individuals may respond differently to frame rates, we also provide subjects of our experiment with interactive controls that pause the animation and step forward or back one frame at a time. We display the frame number in a corner of the animation to further support integration for viewers who wish to count frames that have a given property (e.g., $B > A$) and then estimate the probability.

6.4 Animation vs. Other Encodings of Frequencies

Consider the rate at which the line appears at a specific vertical point in a HOPs animation. Call it the *blink rate*, or the expected number of appearances of the horizontal line in a time period. There is a direct analogy between the blink rate and the encodings used in abstract representations of univariate distributions. Specifically, the width of a violin plot or the color intensity (e.g., saturation or α-blending) of a gradient plot (see Figure 9 and [41]) encode the probability densities of the distribution. HOPs encode these probability densities in the blink rate of the lines at each y-value.

There is a more direct correspondence between HOPs and gradient plots (see Figure 9 in the limiting case. As the frame rate of HOPs reaches the flicker fusion threshold [42], the viewer will begin to perceive the animated HOPs as a single, static abstract representation of the distribution. The displayed animation will "converge" to a gradient plot. A similar effect can be achieved at slower frame rates by adjusting the HOP process slightly so that each frame leaves a trace of the line, which fades slowly. Then, at any point in time, the displayed frame would show multiple blue lines with different intensities. The benefit of the gradient plot is that reading the probability at a *specific* horizontal coordinate is likely to be perceptually easier than reading the probability from the blink rate. Similarly, violin plots encode the probability density for a given horizontal coordinate as line width [43], which is generally considered even easier to decode [16].

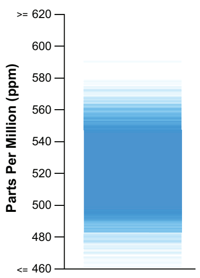

Figure 9. A gradient plot, where probability density of each possible outcome is encoded using mark opacity.

Thus, if the primary task is to find the probability density at a specific point or to find the mean of the distribution, the elevated frame rate may be desirable. The trade-off, however, is that other benefits of HOPs are likely to be eliminated in this "limiting case". Specifically, it will be harder to do direct inference by estimation and counting across frames. We hypothesize that both task

types could be supported by allowing the viewer to interactively manipulate the frame rate (speed up *and* slow down).

6.5 Extensions and Variations of HOPs

Frame Rates & Interactivity—Of course, the speed at which HOPs are presented, and the degree of interactivity allowed will impact the effectiveness of the method, perhaps in different ways for different viewers. While we identified 400ms as a reasonable speed for inferences across distributions with varying reliability levels via informal experiments, an important task for future study is to systematically study the impacts on viewers' accuracy of varying speed of animations and interactive controls.

We suspect that viewers of HOPs could make even more accurate probability inferences if provided with interactive graphical annotations. For example, a viewer might draw a line at the top and bottom of an interval of values that define an outcome of interest to more easily infer the probability of this outcome.

Small Multiples Rather than showing many hypothetical outcome plots as frames in an animation, they could all be displayed simultaneously, as small multiples. In that case, viewers would have to integrate visual information across space rather than across time. Some inferences would be easier. For example, eye gaze could rest longer on frames where values were closer together. On the other hand, comparisons between frames might be harder across spatially distributed multiples than between frames that appear sequentially in the same location sequentially. One important drawback of small multiples is that there is rarely enough space for very many of them. Our HOPs subjects viewed about 90 frames on average for each task (36 seconds if run continuously). It would be visually daunting to see a screen full of 90 very small plots. With many fewer plots, the imprecision due to sampling would be greater, as explored in Section 6.2.

Generating Draws & Stability—Several decisions are particularly critical for HOPs to support accurate inferences. The first important decision involves the integrity of the process used to generate draws from the distribution. The process for generating the hypothetical outcomes should be analogous to the sampling process that resulted in the observed input data set [44]. We refer the analyst interested in apply HOPs to the considerable literature on resampling methods for guidelines on selecting a valid process. The second important decision is how visual stability will be maintained between plots to ensure that they are easily comparable. For more complex plot types, beyond what can be shown in a bar chart, more sophisticated manipulation of the data-to-visual mapping functions may be necessary. Another area to explore is how HOPs frames should be ordered. Presently these are ordered randomly, but other options include minimizing the between frame changes [21], or devising a way of counter-balancing frames.

Combined Representations—It may be useful to combine HOPs with more abstract, static depictions, such that the static display is more fully understood. For example, animated HOPs may be useful in helping people understand the meaning of the interval conveyed by an error bar or area of a violin or gradient plot. HOPs could easily be overlaid on other forms, or enhanced with additional information (e.g., a static median bar).

7 Conclusion

We present and study Hypothetical Outcome Plots as an alternative to static depictions of probability distributions. To create HOPs, we generate draws from a probability distribution and visualize each draw as an outcome plot. When the analyst maintains a consistent mapping function (visual stability) across plots, a viewer can integrate the information from the set of outcomes to make inferences. HOPs facilitate thinking about properties of distributions via counting in addition to probabilities, which is likely to ease data interpretation for many

viewers [5, 6]. HOPs are more expressive of joint distributions than alternative representations like error bars and violin plots.

Avenues for future study of HOPs are numerous. For example, we have only begun to understand the cognitive processes by which viewers integrate information across frames. Additionally, systematic studies of frame rates and interactive capabilities would allow us to better deploy HOPs across diverse datasets and tasks. Finally, maintaining visual stability will be more challenging for more complex types.

Our study results provide strong evidence that HOPs lead to more accurate estimates of properties even of very simple multivariate distributions consisting of just two and three variables, both when the variables are independent or correlated. We also find that HOPs perform substantially worse on univariate judgments only when the variance is high and the task is to estimate the mean. However, we believe that extensions such as the ability to vary the frame rate may support these estimates as well. Other adaptations of HOPs and increased viewer practice may also lead to further improvements in their ability to interpret HOPs. In the long run, the widespread availability of interactive devices will allow visual presentation of uncertainty to shift from its current emphasis on static, abstract representation of probability distributions to dynamic, concrete presentation of hypothetical outcomes from those distributions.

References

1. Taylor BN, Ntnoef C, Taylor BN, Kuyatt CE, Brown RH. Guidelines for evaluating and expressing the uncertainty of NIST measurement results; 1994.

2. Belia S, Fidler F, Williams J, Cumming G. Researchers misunderstand confidence intervals and standard error bars. Psychological methods. 2005;10(4):389.

3. Potter K, Kniss J, Riesenfeld R, Johnson CR. Visualizing summary statistics and uncertainty. In: Proceedings of the 12th Eurographics / IEEE - VGTC conference on Visualization. EuroVis'10. Aire-la-Ville, Switzerland, Switzerland: Eurographics Association; 2010. p. 823–832. Available from: http://dx.doi.org/10.1111/j.1467-8659.2009.01677.x.

4. Correll M, Gleicher M. Error Bars Considered Harmful: Exploring Alternate Encodings for Mean and Error. Visualization and Computer Graphics, IEEE Transactions on. 2014 Dec;20(12):2142–2151.

5. Gigerenzer G, Hoffrage U. How to improve Bayesian reasoning without instruction: Frequency formats. Psychological Review. 1995;102:684–704.

6. Hoffrage U, Gigerenzer G. Using natural frequencies to improve diagnostic inferences. Academic Medicine: Journal of the Association of American Medical Colleges. 1998 May;73(5):538–540.

7. Cumming G, Finch S. Inference by Eye: Confidence Intervals and How to Read Pictures of Data. Am Psychologist. 2005;60(2):170–180.

8. Potter K, Kniss J, Riesenfeld R, Johnson CR. Visualizing Summary Statistics and Uncertainty. Comp Graphics Forum. 2010;29(3):823–832. Available from: http://dx.doi.org/10.1111/j.1467-8659.2009.01677.x.

9. Ibrekk H, Morgan MG. Graphical Communication of Uncertain Quantities to Nontechnical People. Risk Analysis. 1987;7(4):519–529. Available from: http://dx.doi.org/10.1111/j.1539-6924.1987.tb00488.x.

10. Cumming G. Inference by eye: Reading the overlap of independent confidence intervals. Statistics in Medicine. 2009;28(2):205–220. Available from: http://dx.doi.org/10.1002/sim.3471.

11. Newman GE, Scholl BJ. Bar graphs depicting averages are perceptually misinterpreted: The within-the-bar bias. Psychonomic bulletin & review. 2012;19(4):601–607.

12. Bertin J. Semiology of Graphics. University of Wisconsin Press; 1983.

13. Barrowman NJ, Myers RA. Raindrop Plots. The American Statistician. 2003 Nov;57(4):268–274. Available from: http://amstat.tandfonline.com/doi/abs/10.1198/0003130032369.

14. Kampstra P. Beanplot: A Boxplot Alternative for Visual Comparison of Distributions. Journal of Statistical Software, Code Snippets. 2008 11;28(1):1–9. Available from: http://www.jstatsoft.org/v28/c01.

15. Spiegelhalter DJ. Surgical audit: statistical lessons from Nightingale and Codman. Journal of the Royal Statistical Society: Series A (Statistics in Society). 1999 Jan;162(1):45–58. Available from: http://onlinelibrary.wiley.com/doi/10.1111/1467-985X.00120/abstract.

16. Mackinlay J. Automating the Design of Graphical Presentations of Relational Information. ACM Trans Graph. 1986 Apr;5(2):110–141. Available from: http://doi.acm.org/10.1145/22949.22950.

17. Eynon BP, Switzer P. The variability of rainfall acidity. Canadian Journal of Statistics. 1983;11(1):11–23. Available from: http://dx.doi.org/10.2307/3314707.

18. Efron B, Diaconis. Computer-intensive methods in statistics. Scientific American;248(5):116–130.

19. Aerts J, Clarke K, Keuper A. Testing Popular Visualization Techniques for Representing Model Uncertainty. Cartography and Geographic Information Science. 2003 Jul;p. 249–261. Available from: http://dx.doi.org/10.1559/152304003100011180.

20. Bastin L, Fisher PF, Wood J. Visualizing uncertainty in multi-spectral remotely sensed imagery. Computational Geoscience. 2002 Apr;28(3):337–350. Available from: http://dx.doi.org/10.1016/S0098-3004(01)00051-6.

21. Ehlschlaeger CR, Shortridge AM, Goodchild MF. Visualizing spatial data uncertainty using animation. Computers & Geosciences. 1997 May;23(4):387–395. Available from: http://www.sciencedirect.com/science/article/pii/S0098300497000058.

22. Evans BJ. Dynamic display of spatial data-reliability: Does it benefit the map user? Computers & Geosciences. 1997;23(4):409–422.

23. Fisher PF. Visualizing Uncertainty in Soil Maps by Animation. Cartographica: The International Journal for Geographic Information and Geovisualization. 1993 Oct;30(2):20–27. Available from: http://dx.doi.org/10.3138/B204-32P4-263L-76W0.

24. Irwin N, Quealy K. How Not to Be Misled by the Jobs Report. The New York Times. 2014 May;Available from: http://www.nytimes.com/2014/05/02/upshot/how-not-to-be-misled-by-the-jobs-report.html.

25. Bostock M, Carter S, Cox A, Daniel J, Katz J, Quealy K. Who Will Win The Senate? The New York Times. 2014 Apr;Available from: http://www.nytimes.com/newsgraphics/2014/senate-model/#changes.

26. Wickham H, Cook D, Hofmann H, Buja A. Graphical inference for infovis. Visualization and Computer Graphics, IEEE Transactions on. 2010;16(6):973–979.

27. Buja A, Cook D, Hofmann H, Lawrence M, Lee EK, Swayne DF, et al. Statistical inference for exploratory data analysis and model diagnostics. Philosophical transactions Series A, Mathematical, physical, and engineering sciences. 2009 Nov;367(1906):4361–4383.

28. Gelman A. Exploratory data analysis for complex models. Journal of Computational and Graphical Statistics. 2004;13(4).

29. Hofmann H, Follett L, Majumder M, Cook D. Graphical tests for power comparison of competing designs. Visualization and Computer Graphics, IEEE Transactions on. 2012;18(12):2441–2448. Available from: http://ieeexplore.ieee.org/xpls/abs_all.jsp?arnumber=6327249.

30. Majumder M, Hofmann H, Cook D. Validation of visual statistical inference, applied to linear models. Journal of the American Statistical Association. 2013;108(503):942–956.

31. Chance B, Rossman A. Using Simulation to Teach and Learn Statistics;.

32. Cumming G. Understanding The New Statistics: Effect Sizes, Confidence Intervals, and Meta-Analysis. New York: Routledge; 2011.

33. Trick LM, Pylyshyn ZW. Why are small and large numbers enumerated differently? A limited-capacity preattentive stage in vision. Psychological review. 1994;101(1):80.

34. Bostock M, Ogievetsky V, Heer J. D3 Data-Driven Documents. IEEE Transon Visualization and Computer Graphics. 2011 Dec;17(12):2301–2309.

35. Bostock M, Ogievetsky V, Heer J. D3 Data-Driven Documents. IEEE Transactions on Visualization and Computer Graphics. 2011 Dec;17(12):2301–2309. Available from: http://dx.doi.org/10.1109/TVCG.2011.185.

36. Heer J, Bostock M. Crowdsourcing Graphical Perception: Using Mechanical Turk to Assess Visualization Design. In: Proc. of the SIGCHI Conference on Human Factors in Computing Systems. CHI ’10. New York, NY, USA: ACM; 2010. p. 203–212.

37. Ipeirotis P. Demographics of Mechanical Turk: Now Live! (April 2015 edition) | A Computer Scientist in a Business School;. Available from: http://www.behind-the-enemy-lines.com/2015/04/demographics-of-mechanical-turk-now.html.

38. Alvarez GA. Representing multiple objects as an ensemble enhances visual cognition. Trends in cognitive sciences. 2011;15(3):122–131.

39. Albrecht AR, Scholl BJ. Perceptually averaging in a continuous visual world extracting statistical summary representations over time. Psychological Science. 2010;21(4):560–567.

40. Eklund A. beeswarm: The Bee Swarm Plot, an Alternative to Stripchart; 2015. R package version 0.2.0. Available from: http://CRAN.R-project.org/package=beeswarm.

41. Jackson CH. Displaying Uncertainty With Shading. The American Statistician. 2008;62(4):340–347.

42. Schwartz SH, Meese T. Visual perception: A clinical orientation. McGraw-Hill Medical Pub. Division; 2010.

43. Hintze JL, Nelson RD. Violin plots: a box plot-density trace synergism. The American Statistician. 1998;52(2):181–184.

44. Davison AC, Hinkley DV. Bootstrap Methods and their Application. Cambridge University Press; 1997.
