# Supplementary material for: Hypothetical Outcome Plots Outperform Error Bars and Violin Plots for Inferences about Reliability of Variable Ordering
Source: PLoS One. 2015 Nov 16;10(11):e0142444. doi: 10.1371/journal.pone.0142444 (PMC4646698; doi:10.1371/journal.pone.0142444)
Supplement: S2 File — An animated appendix provides more comprehensive results for each question. View the PDF in Adobe Illustrator and click the plots to see the animated version. (PDF) [file pone.0142444.s002.pdf]

Appendix: Hypothetical Outcome Plots Outperform Error Bars and Violin Plots for Inferences About Reliability of Variable Ordering

Jessica Hullman[1,*], Paul Resnick[2], Eytan Adar[2],

1 Information School, University of Washington, Seattle, WA, USA
2 School of Information,University of Michigan, Ann Arbor, MI, USA

*** jhullman@uw.edu**

1 Visualization Stimuli

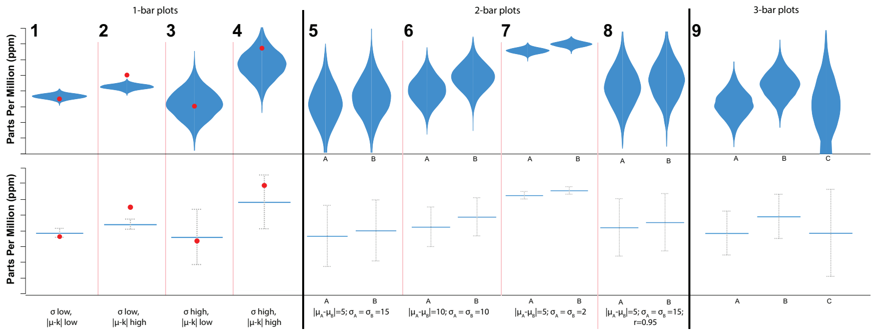

Figure 1. The eight tasks that subjects completed, shown as violin plots and error bars. Click the image to play the HOPs animation.

2 Study Apparatus

Each Mechanical Turk worker who participated in our study completed 4 one-variable plot screens, 4 two-variable plot screens, and 1 three-variable plot screen. An introductory screen preceded the task, and a final screen asked follow-up questions to check understanding.

A testable interface to the study is available here. All interface code, including the software for generating all visualization stimuli, is also included as supplemental materials.

Additionally, an example of each screen is shown below.

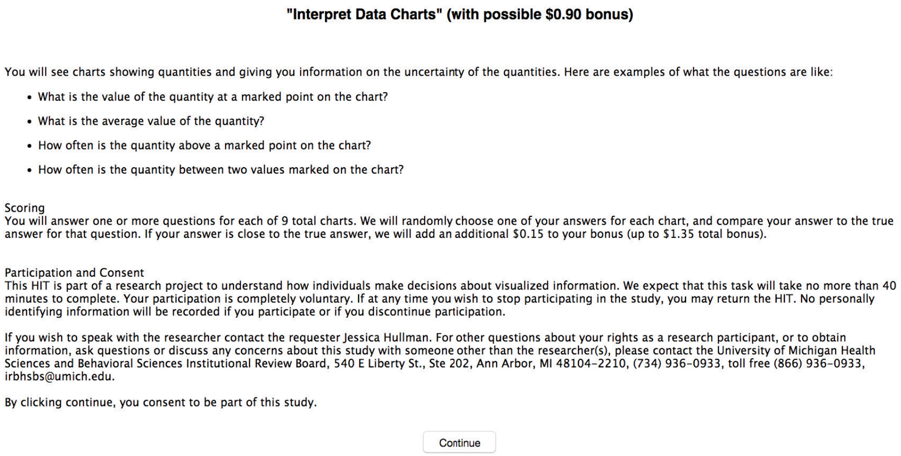

Figure 2. Task introduction screen.

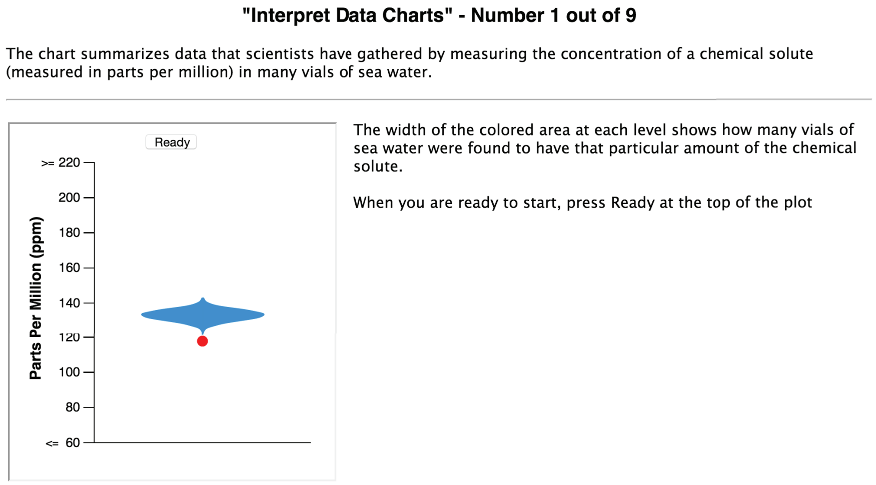

Figure 3. First one-variable screen. Subject must press the 'Ready' button to see the question. In the HOPs treatment, pressing 'Ready' also starts the animation.

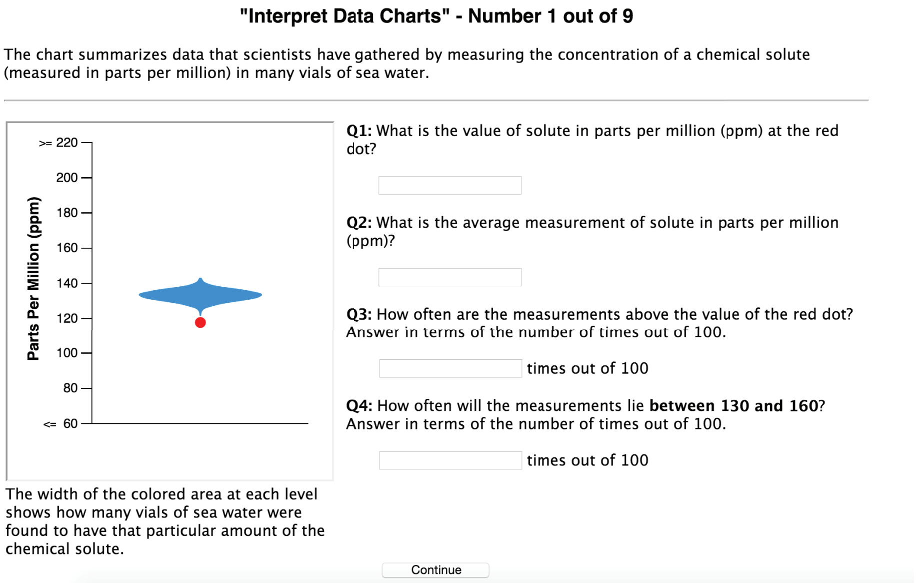

Figure 4. First one-variable screen, after subject has hit ’Ready’ button.

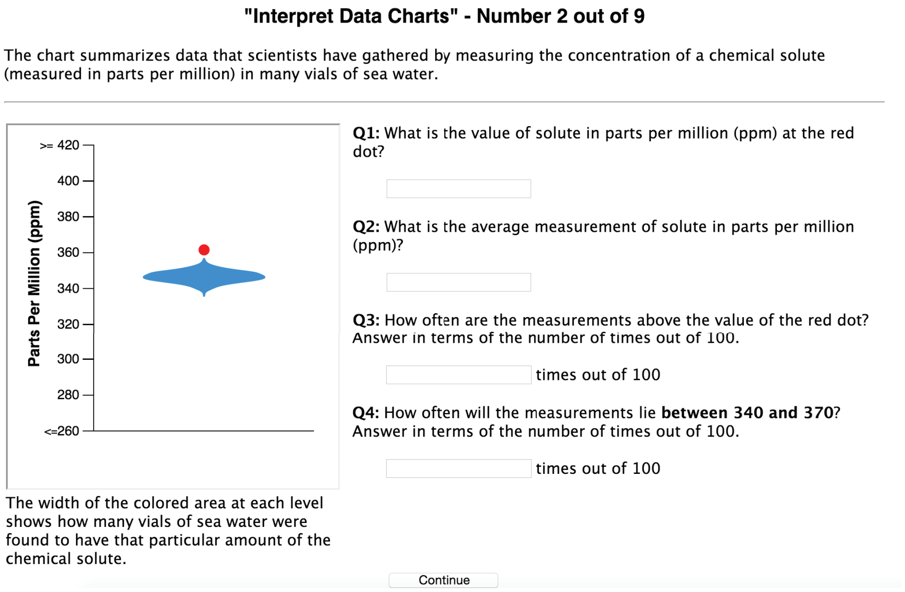

Figure 5. Second one-variable screen.

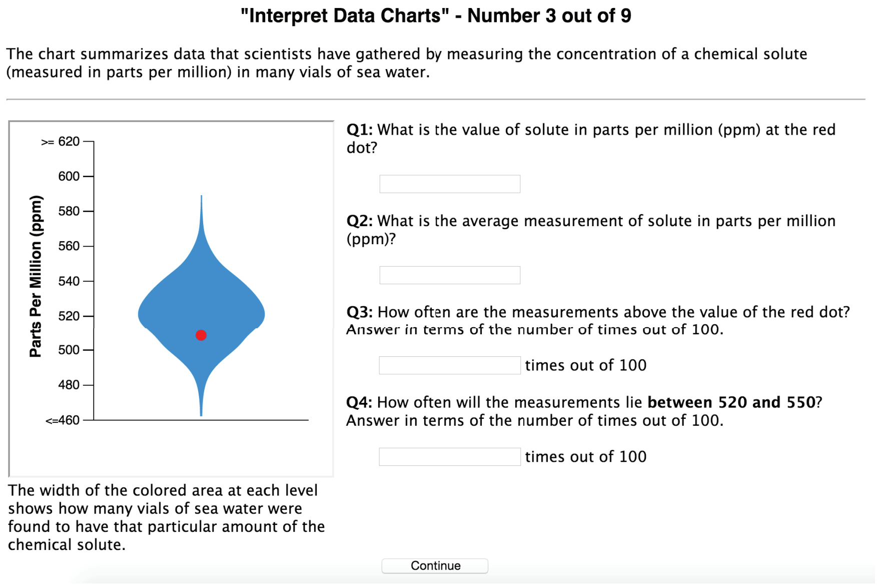

Figure 6. Third one-variable screen.

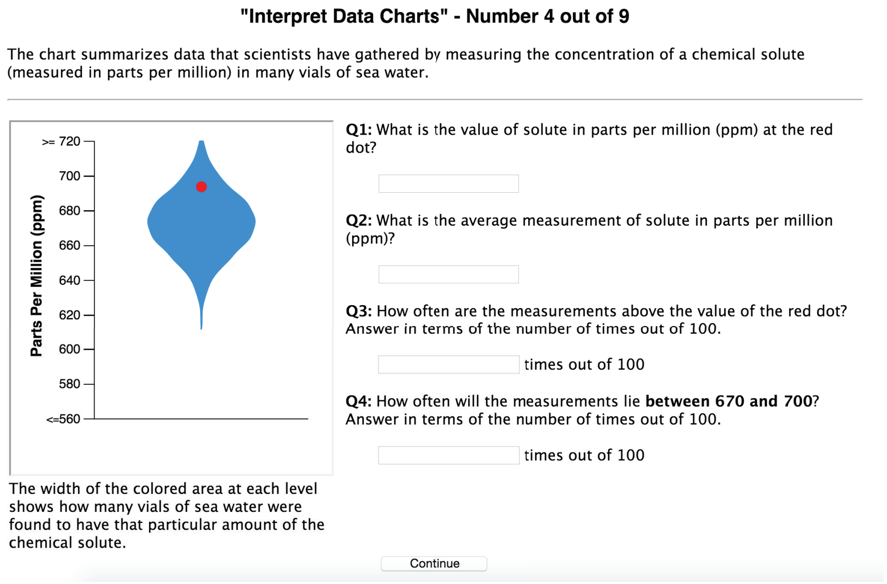

Figure 7. Fourth one-variable screen.

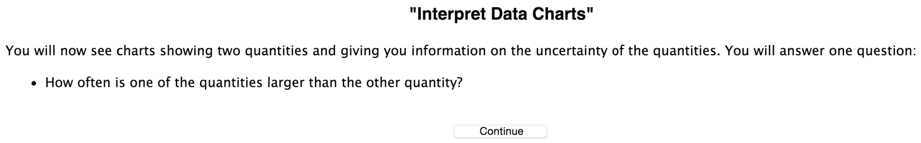

Figure 8. Introduction to two-variable plots.

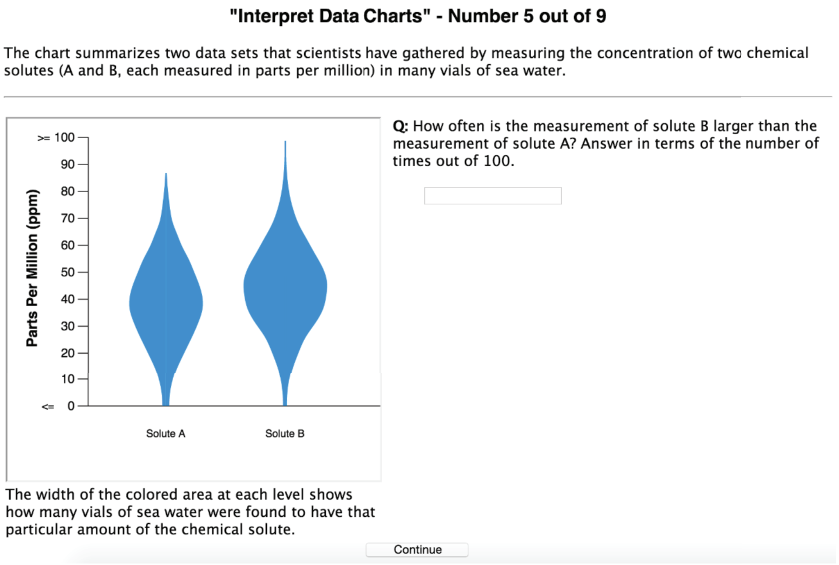

Figure 9. First two-variable screen.

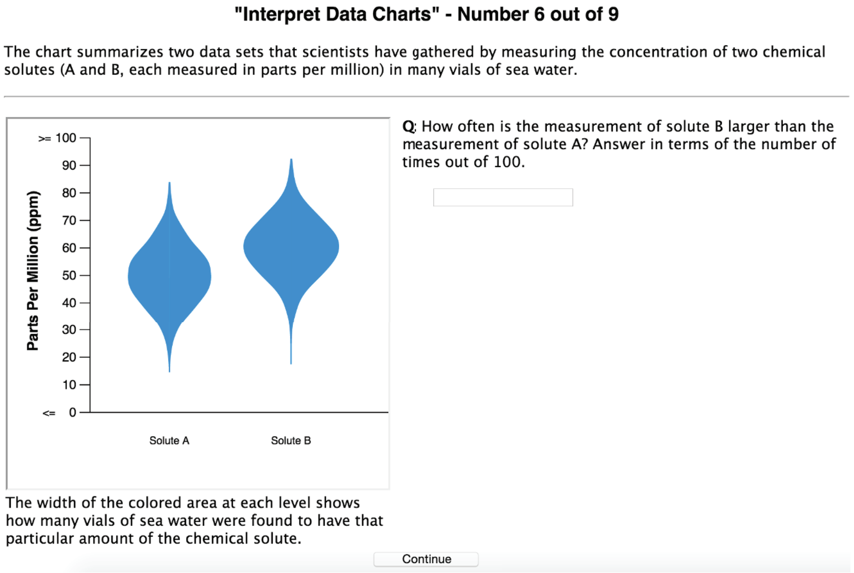

Figure 10. Second two-variable screen.

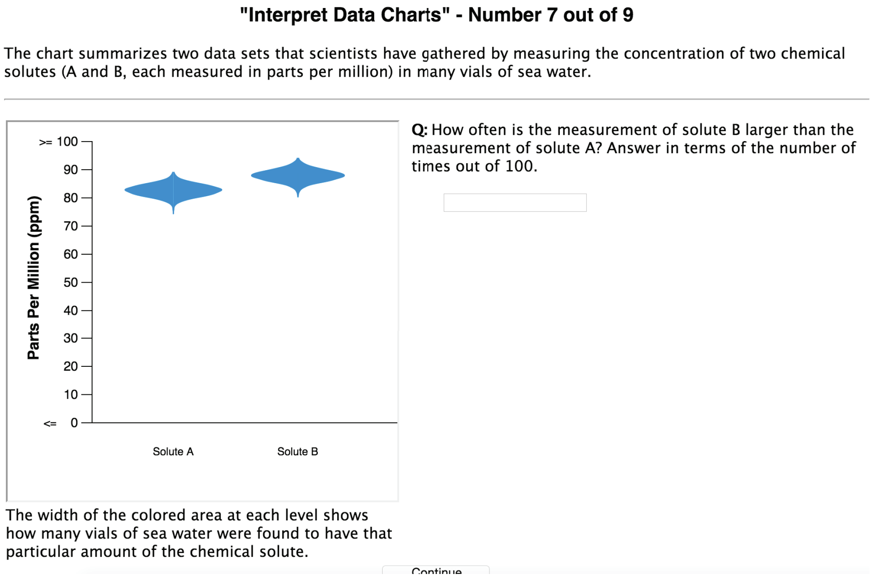

Figure 11. Third two-variable screen.

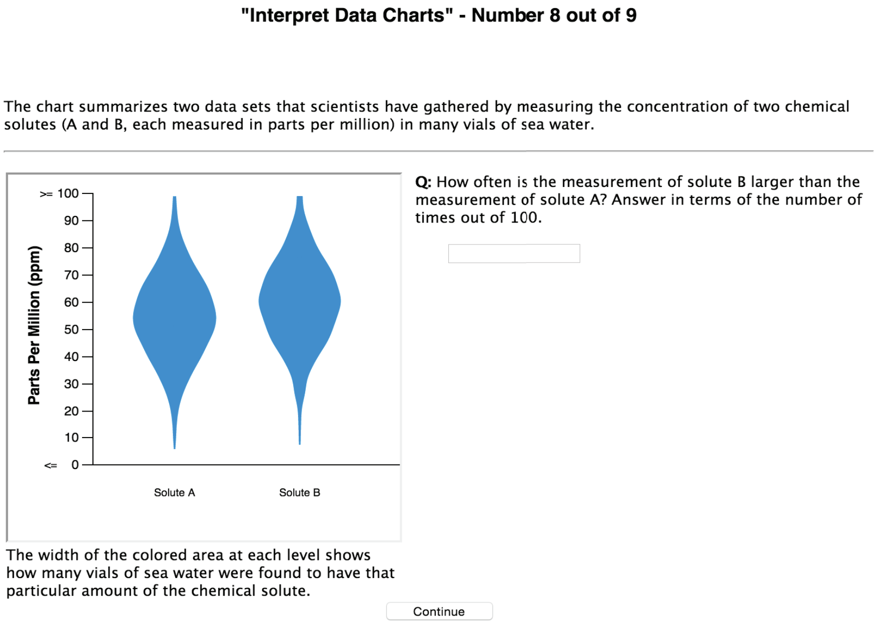

Figure 12. Fourth two-variable screen.

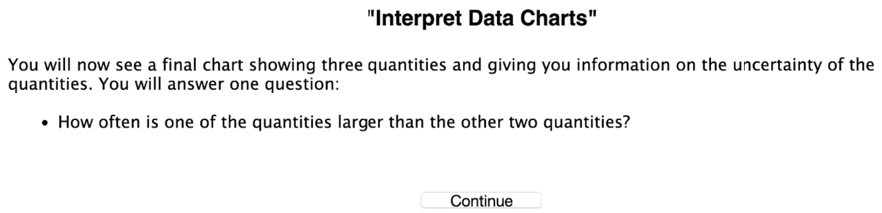

Figure 13. Introduction to three-variable screen.

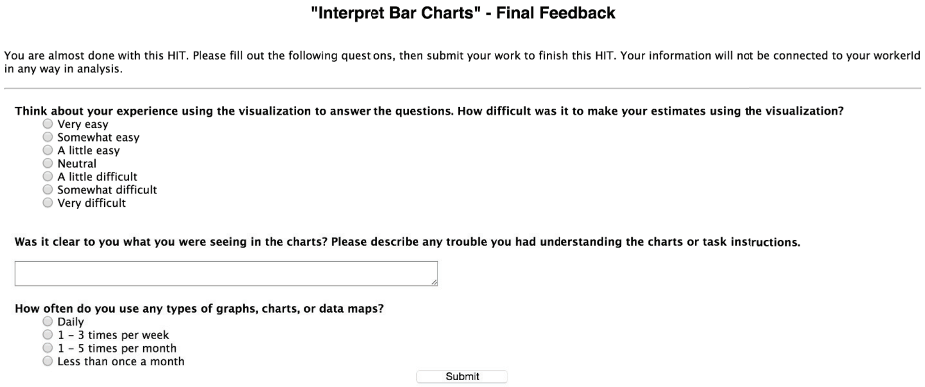

Figure 14. Final screen. Subjects are asked to rate the difficulty of the task and provide feedback on specific difficulties they encountered. Information is gathered about the subject's familiarity with data visualizations.

3 Detailed Results

3.1 One-Variable Plots

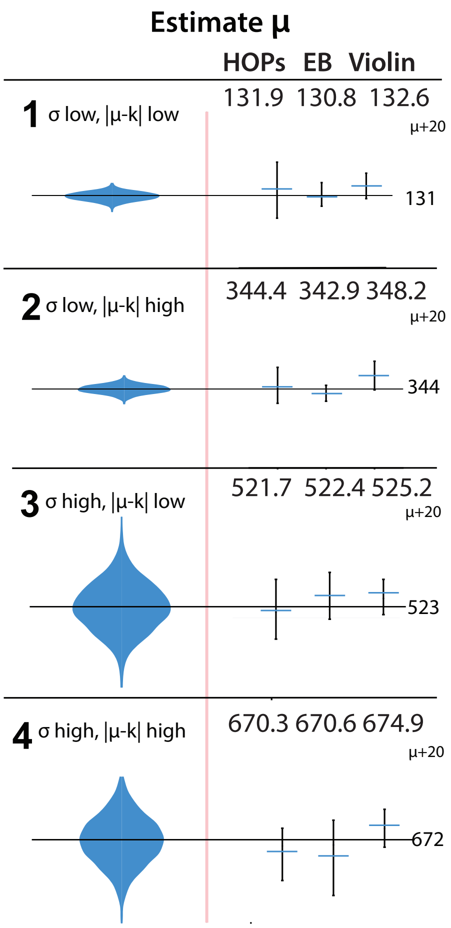

Figure 15. Stimuli (left) and raw responses (right) for estimation of μ.The correct response is marked with a horizontal line. Subjects who used the violin plot consistently overestimated μ. Click the image to play the HOPs animation.

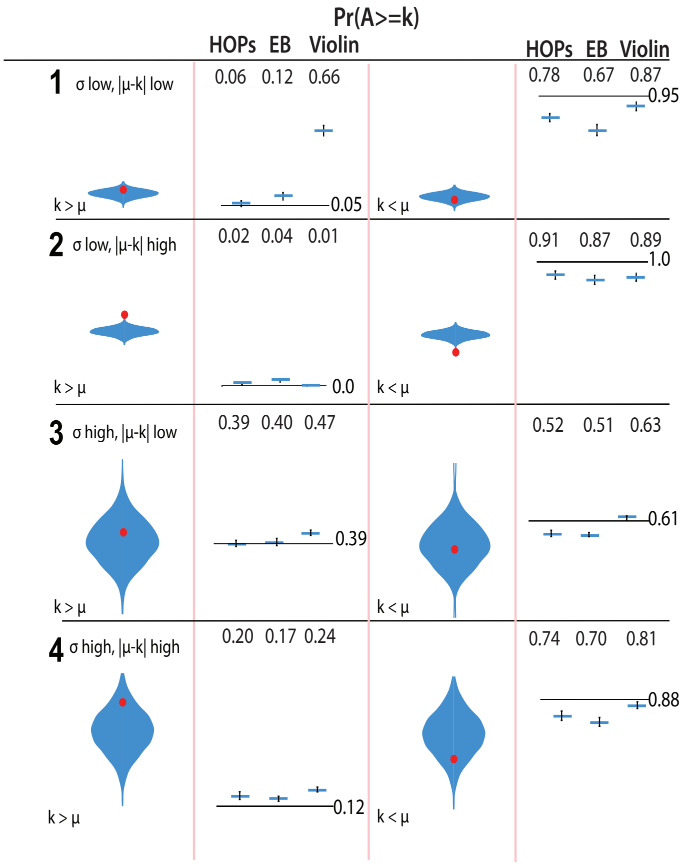

Figure 16. Stimuli (left) and raw responses (right) for estimate of $Pr(A >= k)$. The correct response is marked with a horizontal line. Across all three conditions, subjects show a tendency to under- or overestimate $Pr(A >= k)$ corresponding to the relative position of the red dot. Click the image to play the HOPs animation.

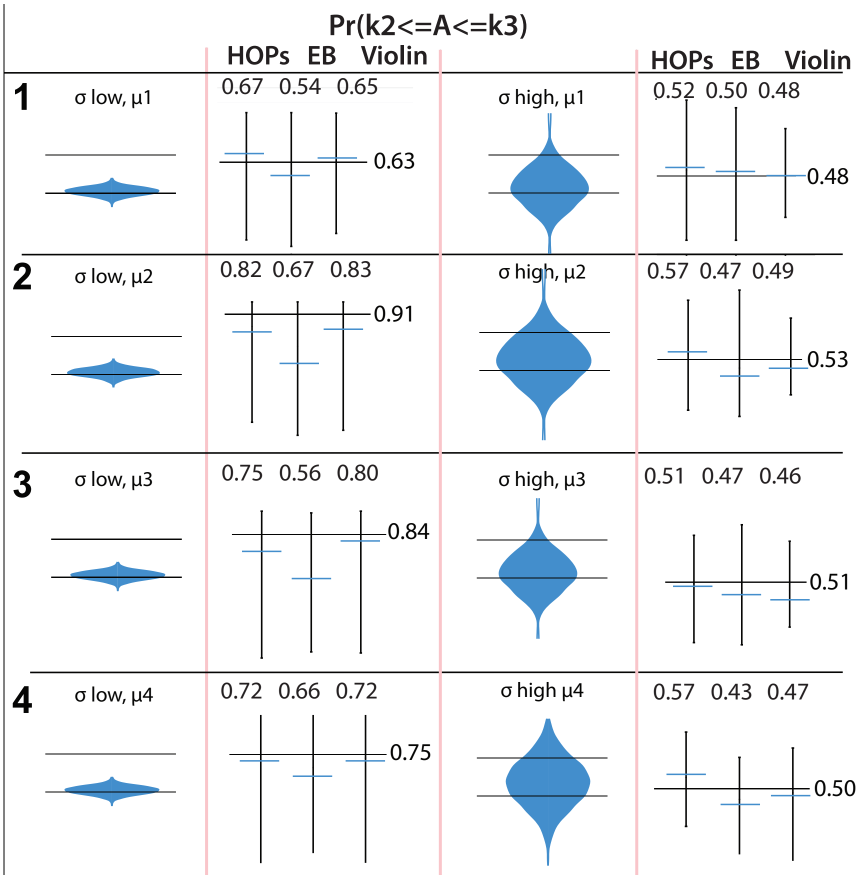

Figure 17. Stimuli (left) and raw responses (right) for estimate of $Pr(k2 <= A <= k3)$. The correct response is marked with a horizontal line. Subjects who used error bars and violin plots show a consistent tendency to underestimate $Pr(k2 <= A <= k3)$. Click the image to play the HOPs animation.

3.2 Two-Variable Tasks

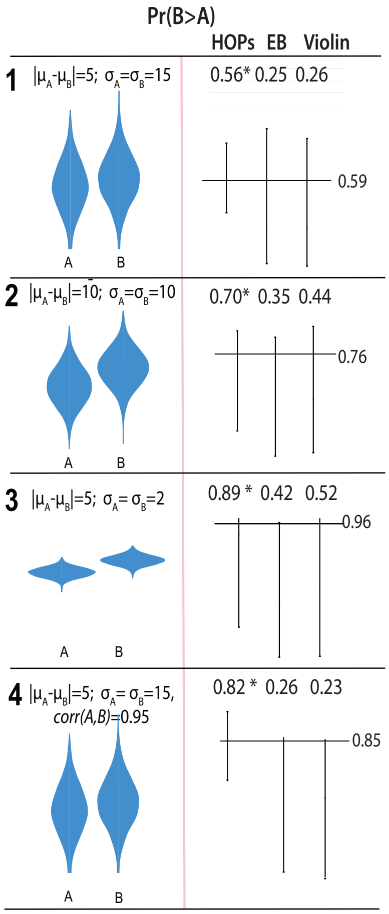

Figure 18. Stimuli (left) and raw responses (right) for estimate of $Pr(B > A)$. The correct response is marked with a horizontal line. With the exception of HOPs subjects for distribution 1, subjects across all three conditions tended to underestimate$Pr(B > A)$. Click the image to play the HOPs animation.

3.3 Three-Variable Task

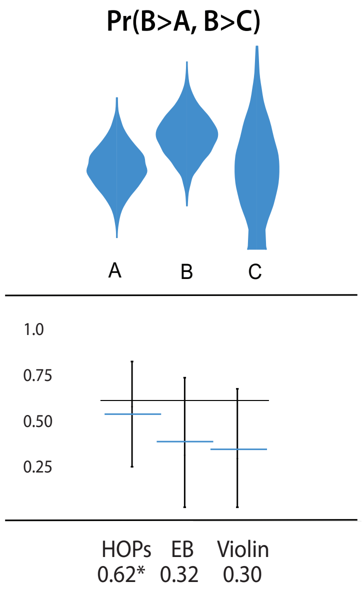

Figure 19. Stimuli (top) and raw responses (bottom) for estimate of $Pr(B > A, B > C)$. The correct response is marked with a horizontal line. Subjects across all three conditions show a consistent tendency to underestimate $Pr(B > A, B > C)$. Click the image to play the HOPs animation.
