# Supplementary material for: Hypothetical Outcome Plots Outperform Error Bars and Violin Plots for Inferences about Reliability of Variable Ordering
Source: PLoS One. 2015 Nov 16;10(11):e0142444. doi: 10.1371/journal.pone.0142444 (PMC4646698; doi:10.1371/journal.pone.0142444)
Supplement: S3 File — A static appendix provides more comprehensive results for each question. (PDF) [file pone.0142444.s003.pdf]

SIGCHI Conference Proceedings Format

1st Author Name
Affiliation
City, Country
e-mail address

2nd Author Name
Affiliation
City, Country
e-mail address

3rd Author Name
Affiliation
City, Country
e-mail address

ABSTRACT

UPDATED—August 16, 2015. This sample paper describes the formatting requirements for SIGCHI conference proceedings, and offers recommendations on writing for the worldwide SIGCHI readership. Please review this document even if you have submitted to SIGCHI conferences before, as some format details have changed relative to previous years. Abstracts should be about 150 words and are required.

Author Keywords

Authors' choice; of terms; separated; by semicolons; commas, within terms only; this section is required.

ACM Classification Keywords

H.5.m. Information Interfaces and Presentation (e.g. HCI): Miscellaneous; See `http://acm.org/about/class/1998/` for the full list of ACM classifiers. This section is required.

INTRODUCTION

This is the intro.

RELATED WORK

This is the related work.

SECTION 1

This is section 1.

SECTION 2

This is section 2.

SECTION 3

This is section 3.

Paste the appropriate copyright statement here. ACM now supports three different copyright statements:
• ACM copyright: ACM holds the copyright on the work. This is the historical approach.
• License: The author(s) retain copyright, but ACM receives an exclusive publication license.
• Open Access: The author(s) wish to pay for the work to be open access. The additional fee must be paid to ACM.
This text field is large enough to hold the appropriate release statement assuming it is single spaced.
Every submission will be assigned their own unique DOI string to be included here.
