# Supplementary material for: Hypothetical Outcome Plots Outperform Error Bars and Violin Plots for Inferences about Reliability of Variable Ordering
Source: PLoS One. 2015 Nov 16;10(11):e0142444. doi: 10.1371/journal.pone.0142444 (PMC4646698; doi:10.1371/journal.pone.0142444)
Supplement: S4 File — All experiment interface and visualization stimuli code is available as a zipped file. (ZIP) [file pone.0142444.s004.zip › experiment_code/final.php]

php
#$workerId = $\_GET['workerId'];
$q1 = $\_POST['q1'];
$timeToRespond = $\_POST['timeToRespond'];
$responseTimes = $\_POST['responseTimes']."::".$timeToRespond;
$naction = $\_POST['naction'];
$actions = $\_POST['actions']."::".$naction;
$q1ans = $\_POST['q1ans'];
$q2ans = $\_POST['q2ans'];
$q3ans = $\_POST['q3ans'];
$q4ans = $\_POST['q4ans'];
$q1ans2 = $\_POST['q1ans2'];
$k0s1 = $\_POST['k0s1'];
$k1s1 = $\_POST['k1s1'];
$k2s1 = $\_POST['k2s1'];
$ends1 = $\_POST['ends1'];
$ends2 = $\_POST['ends2'];
$mytask = $\_POST['mytask'];
$mytask2 = $\_POST['mytask2'];
$treat = $\_POST['treat'];
?


**# "Interpret Bar Charts" - Final Feedback** 
  
  

You are almost done with this HIT. Please fill out the following questions, then submit your work to finish this HIT. Your information will not be connected to your workerId in any way in analysis.   
  


---

  

**Think about your experience using the visualization to answer the questions. How difficult was it to make your estimates using the visualization?**

Very easy  

Somewhat easy  

A little easy  

Neutral  

A little difficult  

Somewhat difficult  

Very difficult  
  
  

**Was it clear to you what you were seeing in the charts? Please describe any trouble you had understanding the charts or task instructions.**

  

  
  

**How often do you use any types of graphs, charts, or data maps?**

Daily  

1 - 3 times per week  

1 - 5 times per month   

Less than once a month

  

### This concludes the experiment. At this point subjects submitted their results.
