# Supplementary material for: Hypothetical Outcome Plots Outperform Error Bars and Violin Plots for Inferences about Reliability of Variable Ordering
Source: PLoS One. 2015 Nov 16;10(11):e0142444. doi: 10.1371/journal.pone.0142444 (PMC4646698; doi:10.1371/journal.pone.0142444)
Supplement: S4 File — All experiment interface and visualization stimuli code is available as a zipped file. (ZIP) [file pone.0142444.s004.zip › experiment_code/index.php]

php
$formAction = "start.php";
?


# Experiment Interface for

  

### This site provides access to the study interface for Hypothetical Outcome Plots Outperform Error Bars and Violin Plots. Choose a visualization treatment below. After clicking Continue you will be presented with the experimental interface for that treatment.

  
Violin Plot  
Error Bar  
HOPs
