# Supplementary material for: Hypothetical Outcome Plots Outperform Error Bars and Violin Plots for Inferences about Reliability of Variable Ordering
Source: PLoS One. 2015 Nov 16;10(11):e0142444. doi: 10.1371/journal.pone.0142444 (PMC4646698; doi:10.1371/journal.pone.0142444)
Supplement: S4 File — All experiment interface and visualization stimuli code is available as a zipped file. (ZIP) [file pone.0142444.s004.zip › experiment_code/question_one.php]

php
$counter = $\_POST['counter'];
$mytask = $\_POST['mytask'];
$treat = $\_POST['treat'];
$orders = explode("::", $\_POST['orders']);
$k0s = explode("::", $\_POST['k0s']);
$k1s = explode("::", $\_POST['k1s']);
$k2s = explode("::", $\_POST['k2s']);
$ends = explode("::", $\_POST['ends']);
$formAction = "./question\_one.php";
//plot code tasks a number (1-96) and a task (1-4)
if($counter  0) { #not the first task
$q1 = $\_POST['q1']; #last answer
$q2 = $\_POST['q2']; #last answer
$q3 = $\_POST['q3']; #last answer
$q4 = $\_POST['q4']; #last answer
$last = $counter - 1;
$naction = $\_POST['naction']; //new actions
$timeToRespond = $\_POST['timeToRespond']; //last response time
if($counter==1) {
$responseTimes = $timeToRespond;
$actions = $naction; #initialize the thing you'll pass each time
$q1ans = $q1;
$q2ans = $q2;
$q3ans = $q3;
$q4ans = $q4;
}else{
$responseTimes = $\_POST['responseTimes']."::".$timeToRespond;
$q1ans = $\_POST['q1ans']."::".$q1;
$q2ans = $\_POST['q2ans']."::".$q2;
$q3ans = $\_POST['q3ans']."::".$q3;
$q4ans = $\_POST['q4ans']."::".$q4;
$actions = $\_POST['actions']."::".$naction;
if($counter == 3) {
$formAction = "./two\_start.php";
}
}
//
}else{
$counter=0;
$myPause = "";
$myForward = "";
$myBack = "";
#if its the first one hide plotFrame, hide question, show plotFrame2
//
}
$num = $counter;
$k0 = $k0s[$counter];
$k1 = $k1s[$counter];
$k2 = $k2s[$counter];
$strends = $ends[$counter];
$arrends = explode("\_", $strends);
#round down/up to nearest 10 for axis range
$e1 = floor($arrends[0]/10)\*10;
$e2 = ceil($arrends[1]/10)\*10;
$orders = implode("::", $orders);
$k0s = implode("::", $k0s);
$k1s = implode("::", $k1s);
$k2s = implode("::", $k2s);
$ends = implode("::", $ends);
$counter = $counter+1;
$qnum = $counter;
$patt = '/violin/';
$patt2 = '/chops/';
$patt3 = '/eb/';
preg\_match($patt, $treat, $matches);
preg\_match($patt2, $treat, $matches2);
preg\_match($patt3, $treat, $matches3);
#echo "mytask: ".$mytask."  
";
#echo "e1: ".$e1."  
";
#echo "e2: ".$e2."  
";
#echo "actions: ".$actions."  
";
$feedback = "";
if($matches) {
$frameSrc = "./violin\_one.php?task=".$mytask."&k0=".$k0."#=".$num."&e1=".$e1."&e2=".$e2;
#$frameSrc = "./violin\_one.php?filen=".$filen."&lab=".$qnum;
$plotFrame = "The width of the colored area at each level shows how many vials of sea water were found to have that particular amount of the chemical solute.";
$plotFrame2 = "When you are ready to start, press Ready at the top of the plot";
}else if($matches2) {
$feedback = "";
$frameSrc = "./dot\_chops\_one.php?task=".$mytask."&k0=".$k0."#=".$num."&e1=".$e1."&e2=".$e2;
$plotFrame = "Each plot shows the quantity of solute in one vial of seawater. **Use the buttons at the top of the plot to pause (), play (),  
or step forward () and back () through the plots** if you want to see them at your own pace.";
$plotFrame2 = "When you are ready to start, press play () at the top of the plot";
}else if($matches3) {
$frameSrc = "./dot\_one\_eb.php?task=".$mytask."&k0=".$k0."#=".$num."&e1=".$e1."&e2=".$e2;
$plotFrame = "The blue line shows the average amount of solute in all the seawater vials. The dashed lines show an error bar, a range above and below the average. 95% of the collected vials fall in the range defined by the dashed lines.";
$plotFrame2 = "When you are ready to start, press Ready at the top of the plot";
}else{
$frameSrc = "./dot\_one\_eb.php?task=".$mytask."&k0=".$k0."#=".$num."&e1=".$e1."&e2=".$e2;
}
#$formAction = "https://workersandbox.mturk.com/mturk/externalSubmit";
?>


**# "Interpret Data Charts" - Number php echo $qnum; ? out of 9** 
  

The chart summarizes data that scientists have gathered by measuring the concentration of a chemical solute (measured in parts per million) in many vials of sea water. php echo $feedback; ?

  


---

  

|  |  |
| --- | --- |
| php echo $plotFrame; ? | php echo $plotFrame; ?  php echo $plotFrame2; ?  **Q1:** What is the value of solute in parts per million (ppm) at the red dot?       **Q2:** What is the average measurement of solute in parts per million (ppm)?       **Q3:** How often are the measurements above the value of the red dot? Answer in terms of the number of times out of 100.    times out of 100    **Q4:** How often will the measurements lie **between php echo $k1; ? and php echo $k2; ?**? Answer in terms of the number of times out of 100.    times out of 100 |
