# Supplementary material for: Hypothetical Outcome Plots Outperform Error Bars and Violin Plots for Inferences about Reliability of Variable Ordering
Source: PLoS One. 2015 Nov 16;10(11):e0142444. doi: 10.1371/journal.pone.0142444 (PMC4646698; doi:10.1371/journal.pone.0142444)
Supplement: S4 File — All experiment interface and visualization stimuli code is available as a zipped file. (ZIP) [file pone.0142444.s004.zip › experiment_code/question_two.php]

php
#stuff from one quant task
$q1ans = $\_POST['q1ans'];
$q2ans = $\_POST['q2ans'];
$q3ans = $\_POST['q3ans'];
$q4ans = $\_POST['q4ans'];
$k0s1 = $\_POST['k0s1'];
$k1s1 = $\_POST['k1s1'];
$k2s1 = $\_POST['k2s1'];
$ends1 = $\_POST['ends1'];
$mytask = $\_POST['mytask'];
#stuff from two quant task
$counter = $\_POST['counter'];
$mytask2 = $\_POST['mytask2'];
#echo "$treat $mytask (one quant), $mytask2 (two quant)<br";
$treat = $\_POST['treat'];
$ends = explode("::", $\_POST['ends']);
$feedback= "";
$naction = $\_POST['naction']; #last action
$timeToRespond = $\_POST['timeToRespond'];
$formAction = "./question\_two.php";
if($counter > 0) { #not the first task
$responseTimes = $\_POST['responseTimes']."::".$timeToRespond;
$actions = $\_POST['actions']."::".$naction;
$q1 = $\_POST['q1']; #last answer
$last = $counter - 1;
if($counter==1) {
$q1ans2 = $q1;
}else{
$q1ans2 = $\_POST['q1ans2']."::".$q1;
$actions = $\_POST['actions']."::".$naction;
if($counter == 3) {
$feedback = "This task was included in the experiment, but omitted from reported results since A and B are correlated and this information is not available in the violin plots or error bars.  
";
$formAction = "./three\_start.php";
}
}
}else{
$counter=0;
$actions = $\_POST['actions'];
$responseTimes = $\_POST['responseTimes'];
}
$num = $counter;
$e1 = 0;
$e2 = 100;
$ends = implode("::", $ends);
$counter = $counter+1;
$qnum = $counter+4;
$patt = '/violin/';
$patt2 = '/chops/';
$patt3 = '/eb/';
preg\_match($patt, $treat, $matches);
preg\_match($patt2, $treat, $matches2);
preg\_match($patt3, $treat, $matches3);
if($matches) {
$frameSrc = "./violin\_two.php?task=".$mytask2."#=".$num."&e1=".$e1."&e2=".$e2;
#$frameSrc = "./violin\_one.php?filen=".$filen."&lab=".$qnum;
$plotFrame = "The width of the colored area at each level shows how many vials of sea water were found to have that particular amount of the chemical solute.";
}else if($matches2) {
$frameSrc = "./dot\_chops\_two.php?task=".$mytask2."#=".$num."&e1=".$e1."&e2=".$e2;
$plotFrame = "Each plot shows the quantity of solute in one vial of seawater for each of A and B. **Use the buttons at the top of the plot to pause, restart, or step forward and back through the plots** if you want to see them at your own pace.";
}else if($matches3) {
$frameSrc = "./dot\_two\_eb.php?task=".$mytask2."#=".$num."&e1=".$e1."&e2=".$e2;
$plotFrame = "The blue lines show the average amounts of solute A and B in all the seawater samples. The dashed lines show error bars, ranges above and below the averages. 95% of the collected samples fall in the ranges defined by the dashed lines.";
}else{
$frameSrc = "./dot\_two\_eb.php?task=".$mytask2."#=".$num."&e1=".$e1."&e2=".$e2;
}
#echo "actions: ".$actions."  
";
#$formAction = "https://workersandbox.mturk.com/mturk/externalSubmit";
?>


**# "Interpret Data Charts" - Number php echo $qnum; ? out of 9** 
  
php echo $feedback;?
  

The chart summarizes two data sets that scientists have gathered by measuring the concentration of two chemical solutes (A and B, each measured in parts per million) in many vials of sea water.

  


---

  

|  |  |
| --- | --- |
| php echo $plotFrame; ? | **Q:** How often is the measurement of solute B larger than the measurement of solute A? Answer in terms of the number of times out of 100. |
