# Supplementary material for: Hypothetical Outcome Plots Outperform Error Bars and Violin Plots for Inferences about Reliability of Variable Ordering
Source: PLoS One. 2015 Nov 16;10(11):e0142444. doi: 10.1371/journal.pone.0142444 (PMC4646698; doi:10.1371/journal.pone.0142444)
Supplement: S4 File — All experiment interface and visualization stimuli code is available as a zipped file. (ZIP) [file pone.0142444.s004.zip › experiment_code/start.php]

php
$treat = $\_POST['treat'];
$feedback = "";
if(is\_null($treat)) {
$feedback = "<font color='red'You did not choose a visualization treatment. The HOPs treatment has been loaded.";
$treat = "chops";
}
$mytask = 1;
$parfile = "./data/one/parameters.csv";
$fh = fopen($parfile, 'r');
$theData = fread($fh, filesize($parfile));
$order\_array = array();
$k0\_array = array();
$k1\_array = array();
$k2\_array = array();
$mu\_array = array();
$end\_array = array();
$data\_array = explode("\n", $theData);
fclose($fh);
$line = $data\_array[$mytask];
#echo "taskline ".$line."  
";
#split the line
$tmp = explode(",", $line);
// #first, figure out the order they see - task\_1\_type, etc.
// $order\_array = [$tmp[2],$tmp[5],$tmp[8],$tmp[11]];
// #create an array for all the paramters -- not yet ordered according to their task order
#k1\_1, k1\_2, etc - the red dot
$k0\_array = [$tmp[27],$tmp[29],$tmp[31],$tmp[33]];
#start of range that we ask them about
$k1\_array = [$tmp[18],$tmp[19],$tmp[20],$tmp[21]];
#end of range that we ask them about
$k2\_array = [$tmp[22],$tmp[23],$tmp[24],$tmp[25]];
#start and end points of y axis - always have range of 100, starting at a multiple of 100.
function ceil100($number)
{
return ceil( $number / 100 ) \* 100 + 20;
}
function floor100($number)
{
return floor( $number / 100 ) \* 100 - 40;
}
$end\_array = [floor100($tmp[14])."\_".ceil100($tmp[14]), floor100($tmp[15])."\_".ceil100($tmp[15]),floor100($tmp[16])."\_".ceil100($tmp[16]),floor100($tmp[17])."\_".ceil100($tmp[17])];
$k0s = implode("::", $k0\_array);
$k1s = implode("::", $k1\_array);
$k2s = implode("::", $k2\_array);
$ends = implode("::", $end\_array);
$formAction = "question\_one.php";
$counter = 0;
?>


# "Interpret Data Charts" (with possible $0.90 bonus)

  
php echo $feedback; ?
  
  

### You will see charts showing quantities and giving you information on the uncertainty of the quantities. Here are examples of what the questions are like: - What is the value of the quantity at a marked point on the chart? - What is the average value of the quantity? - How often is the quantity above a marked point on the chart? - How often is the quantity between two values marked on the chart? Scoring You will answer one or more questions for each of 9 total charts. We will randomly choose one of your answers for each chart, and compare your answer to the true answer for that question. If your answer is close to the true answer, we will add an additional $0.15 to your bonus (up to $1.35 total bonus). Participation and Consent This HIT is part of a research project to understand how individuals make decisions about visualized information. We expect that this task will take no more than 40 minutes to complete. Your participation is completely voluntary. If at any time you wish to stop participating in the study, you may return the HIT. No personally identifying information will be recorded if you participate or if you discontinue participation. If you wish to speak with the researcher contact the requester Jessica Hullman. For other questions about your rights as a research participant, or to obtain information, ask questions or discuss any concerns about this study with someone other than the researcher(s), please contact the University of Michigan Health Sciences and Behavioral Sciences Institutional Review Board, 540 E Liberty St., Ste 202, Ann Arbor, MI 48104-2210, (734) 936-0933, toll free (866) 936-0933, irbhsbs@umich.edu. By clicking continue, you consent to be part of this study.
