# Supplementary material for: Hypothetical Outcome Plots Outperform Error Bars and Violin Plots for Inferences about Reliability of Variable Ordering
Source: PLoS One. 2015 Nov 16;10(11):e0142444. doi: 10.1371/journal.pone.0142444 (PMC4646698; doi:10.1371/journal.pone.0142444)
Supplement: S4 File — All experiment interface and visualization stimuli code is available as a zipped file. (ZIP) [file pone.0142444.s004.zip › experiment_code/three_start.php]

php
#stuff from one quant task
$q1ans = $\_POST['q1ans'];
$q2ans = $\_POST['q2ans'];
$q3ans = $\_POST['q3ans'];
$q4ans = $\_POST['q4ans'];
$q1ans2 = $\_POST['q1ans2'];
$k0s1 = $\_POST['k0s1'];
$k1s1 = $\_POST['k1s1'];
$k2s1 = $\_POST['k2s1'];
$ends1 = $\_POST['ends1'];
$ends2 = $\_POST['ends'];
$mytask = $\_POST['mytask'];
#stuff from two quant task
$mytask2 = $\_POST['mytask2'];
$treat = $\_POST['treat'];
$myPause = $\_POST['myPause'];
$myForward = $\_POST['myForward'];
$myBack = $\_POST['myBack'];
$formAction = "./final.php";
$q1 = $\_POST['q1']; #last answer for two quant task
$naction = $\_POST['naction']; #last action
$actions = $\_POST['actions']."::".$naction; #last action
$q1ans2 = $\_POST['q1ans2']."::".$q1;
$formAction = "./question\_three.php";
$timeToRespond = $\_POST['timeToRespond'];
$responseTimes = $\_POST['responseTimes']."::".$timeToRespond;
?


# "Interpret Data Charts"


### You will now see a final chart showing three quantities and giving you information on the uncertainty of the quantities. You will answer one question: - How often is one of the quantities larger than the other two quantities?
