# Supplementary material for: Hypothetical Outcome Plots Outperform Error Bars and Violin Plots for Inferences about Reliability of Variable Ordering
Source: PLoS One. 2015 Nov 16;10(11):e0142444. doi: 10.1371/journal.pone.0142444 (PMC4646698; doi:10.1371/journal.pone.0142444)
Supplement: S4 File — All experiment interface and visualization stimuli code is available as a zipped file. (ZIP) [file pone.0142444.s004.zip › experiment_code/two_start.php]

php
$timeToRespond = $\_POST['timeToRespond'];
$responseTimes = $\_POST['responseTimes']."::".$timeToRespond;
#echo $workerId;
$mytask = $\_POST['mytask'];
$myPause = $\_POST['myPause'];
$naction = $\_POST['naction'];
$actions = $\_POST['actions']."::".$naction;
$myfile = $\_POST['myfile'];
#myfile is datetime\_treat\_mytask (1-96)
$treat = $\_POST['treat'];
#first task params
$k0s1 = $\_POST['k0s']; #last answer
$k1s1 = $\_POST['k1s']; #last answer
$k2s1 = $\_POST['k2s']; #last answer
$ends1 = $\_POST['ends']; #last answer
$q1 = $\_POST['q1']; #last answer
$q2 = $\_POST['q2']; #last answer
$q3 = $\_POST['q3']; #last answer
$q4 = $\_POST['q4']; #last answer
$q1ans = $\_POST['q1ans']."::".$q1;
$q2ans = $\_POST['q2ans']."::".$q2;
$q3ans = $\_POST['q3ans']."::".$q3;
$q4ans = $\_POST['q4ans']."::".$q4;
#echo "$treat $mytask (one quant), $mytask2 (two quant)<br";
$last = 3;
#two quantity task
#Take the subject role (1-96) and take the remainder when dividing by 24. That will give you the row number to use from the parameters file (and the .csv file to use).
#For all four tasks, the y-axis range should be 0-100.
#find the task number for two quantity tasks by remainder after dividing by 24
$mytask2 = $mytask % 24;
if($mytask2==0) {
$mytask2=24;
}
#echo "2 quant task num: ".$mytask2."  
";
#fort his one, you don't need anything from parameters.
#y axis range is always 0 and 100
$end\_array= ["0\_100","0\_100","0\_100","0\_100"];
$ends = implode("::", $end\_array);
$formAction = "question\_two.php";
?>


# "Interpret Data Charts"


### You will now see charts showing two quantities and giving you information on the uncertainty of the quantities. You will answer one question: - How often is one of the quantities larger than the other quantity?
