# Supplementary material for: Hypothetical Outcome Plots Outperform Error Bars and Violin Plots for Inferences about Reliability of Variable Ordering
Source: PLoS One. 2015 Nov 16;10(11):e0142444. doi: 10.1371/journal.pone.0142444 (PMC4646698; doi:10.1371/journal.pone.0142444)
Supplement: S4 File — All experiment interface and visualization stimuli code is available as a zipped file. (ZIP) [file pone.0142444.s004.zip › experiment_code/violin_three.php]

php
$task = $\_GET['task'];
$num = $\_GET['num'];
$e1 = $\_GET['e1'];
$e2 = $\_GET['e2'];
$filen = "./data/three/".$task.".js";
# echo "Provide three URL parameters: mean={15, 20, ... 80}, sd={30, 40 ... 210}, and potent={mean +/- 5, 10 or 15}";
?
